# Gut microbiota–immune crosstalk in childhood and adolescent obesity: mechanistic insights and therapeutic potential focused on short-chain fatty acids and emerging metabolites

**DOI:** 10.3389/fmicb.2026.1904757

**Published:** 2026-07-29

**Authors:** Xingmeng Song, Qiongyue Zhang, Ahmad Khan, Jing Shang, Jianjiang Xue

**Affiliations:** 1 University-Town Hospital of Chongqing Medical University, Chongqing, China; 2 Department of Occupational and Environmental Health, School of Public Health, Chongqing Medical University, Chongqing, China; 3 Oncology Hematology Department, University-Town Hospital of Chongqing Medical University, Chongqing, China; 4 Department of Clinical Laboratory, University-Town Hospital of Chongqing Medical University, Chongqing, China

**Keywords:** childhood obesity, gut microbiota, immune–metabolic crosstalk, novel microbial metabolites, short-chain fatty acids

## Abstract

Childhood and adolescent obesity should not be explained only as a problem of eating too much energy and using too little energy. Research now shows that obesity in young people is also linked to changes in gut bacteria, changes in the way the body uses energy, and long-term mild inflammation during key stages of growth. Acting as a central metabolic nexus, the gut microbiota converts dietary substrates into short-chain fatty acids (SCFAs) along with a broad spectrum of other functional metabolites that shape the microbiota–immune–obesity axis. This review focuses on how SCFAs affect intestinal immunity, how they may be involved in innate immune training, and how they may help promote heat production in fat tissue. These effects are mainly related to the activation of cell receptors called G protein-coupled receptors (GPCRs) and the inhibition of histone deacetylase (HDAC). We further clarify the contributions of several emerging classes of metabolites: aromatic amino acid (AAA) catabolites that affect intestinal barrier integrity and mucosal inflammation; secondary bile acids that modulate energy metabolism through farnesoid X receptor (FXR) and Takeda G protein-coupled receptor 5 (TGR5); and N-acyl amides that act on endocannabinoid-like pathways to adjust appetite. Based on these insights, we propose a three-level intervention strategy focused on microbial metabolites—upstream reshaping of beneficial communities using prebiotics and high-fiber dietary patterns, midstream development of pediatric-specific SCFA formulations and targeted metabolite supplements, and downstream exploration of receptor-targeted pharmacotherapy. Future advances rely on integrated multi-omics designs, rigorous causal evidence from human studies, and development-appropriate personalized regimens, which will ultimately create new precision pathways for the prevention and treatment of early-life obesity.

## 1 Introduction

The reciprocal crosstalk between gut microbiota and the host relies predominantly on diet-derived bioactive metabolites. These metabolites can act as signaling molecules that influence intestinal immune activity, systemic energy metabolism, and inflammatory responses (Bäckhed et al., 2005; Zhou et al., 2023). In childhood and adolescent obesity, alterations in gut microbial composition and function can reshape the type and abundance of microbial metabolites, particularly short-chain fatty acids (SCFAs), bile acid derivatives, and amino acid catabolites (Jiang et al., 2025). These changes may link gut microbial imbalance with long-term mild inflammation and problems in whole-body metabolism (Zhang and Dang, 2022; Yu Z. J. et al., 2023; Cho, 2023). Therefore, this review focuses on the “metabolite–microbiota–immune” axis to better understand how pediatric obesity develops and how future interventions may be designed based on biological mechanisms.

### 1.1 Reconsidering childhood and adolescent obesity: puberty as an immune-metabolic susceptibility window beyond energy imbalance

**Figure 1:**
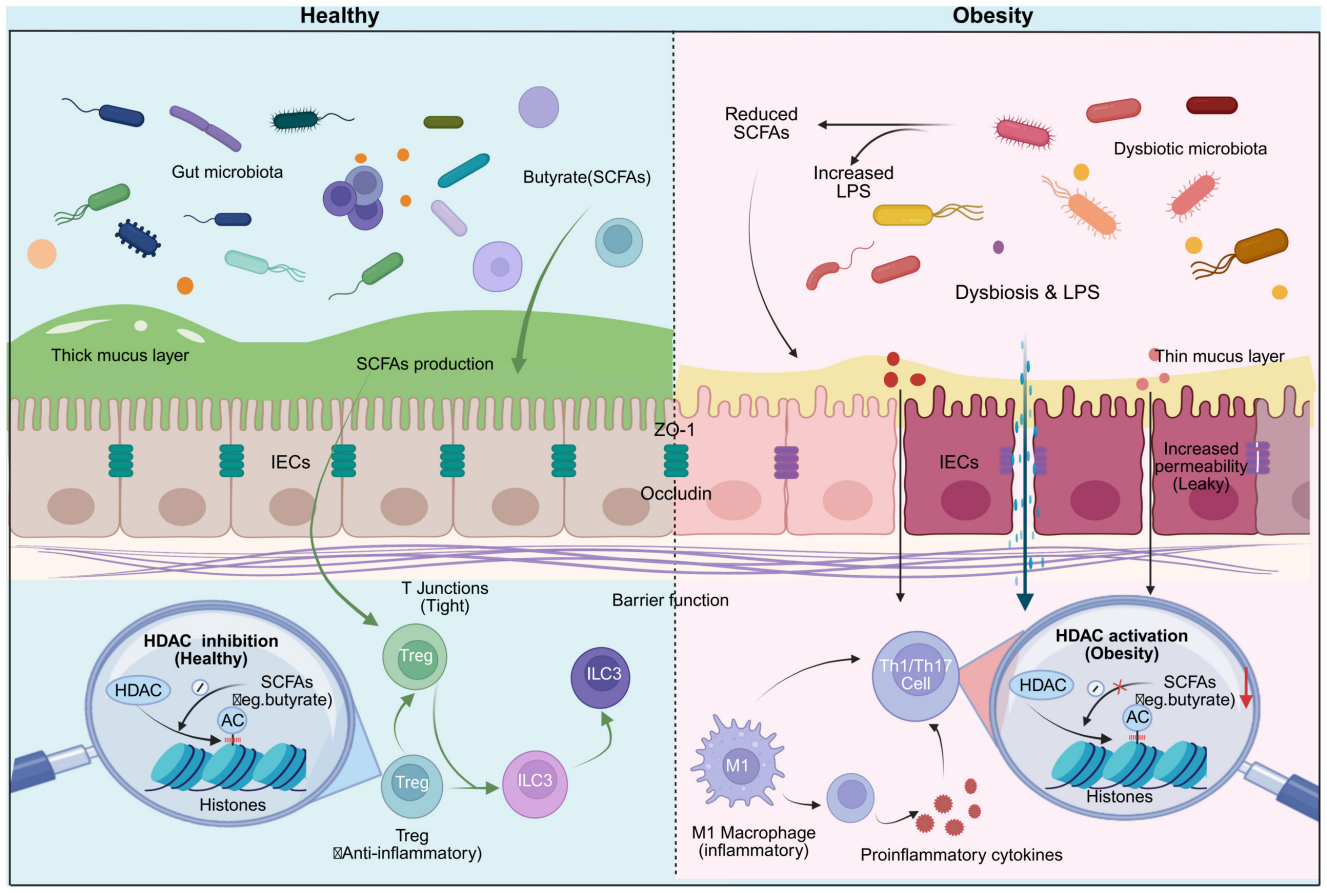
Comparison of gut microbiota, gut barrier, and immune balance between healthy and obese conditions. Created in BioRender. Qy, Z. (2026). https://BioRender.com/qmvi4kv.

Childhood obesity has often been explained as the result of taking in more energy than the body uses (Baranowski and Motil, 2021; Verduci et al., 2021a). However, this energy-balance model alone cannot fully explain why obesity during childhood and adolescence is strongly associated with persistent low-grade inflammation, insulin resistance, pubertal abnormalities, and later metabolic complications (Cho, 2023; Mindru et al., 2025). A more development-oriented interpretation is therefore needed, in which pediatric obesity is viewed not only as excessive fat accumulation but also as an immune-metabolic disorder occurring during a period of rapid growth and physiological remodeling. Puberty represents a particularly sensitive window for obesity-related immune and metabolic dysregulation. During this stage, sex hormones, growth hormone, insulin sensitivity, adipose tissue distribution, and immune maturation change rapidly (Huang and Roth, 2021; Carson et al., 2023). These developmental changes may amplify the inflammatory consequences of excess adiposity, making adolescents more vulnerable to chronic low-grade inflammation than would be predicted by body weight alone. In this context, gut microbiota and microbial metabolites should be understood as modulators of pubertal inflammatory susceptibility rather than as isolated descriptive markers. During childhood and adolescence, the gut microbiota and the immune system are still developing at the same time. Diet, daily habits, and environmental factors, such as antibiotic use and not getting enough sleep, can easily affect the balance of gut bacteria. These factors may also change the metabolite environment in the gut and lead to immune and inflammatory responses (Vallianou et al., 2021; Gunawan et al., 2024). Several pediatric cohorts have reported reduced microbial diversity and altered abundances of beneficial bacteria, including *Akkermansia muciniphila*, and *Faecalibacterium prausnitzii*, in children and adolescents with obesity. However, these findings are not consistent across all populations, and the direction or magnitude of these microbial changes may vary according to ethnicity, geography, diet, age, pubertal status, metabolic phenotype, and sequencing methodology (de Cuevillas et al., 2022; Yuan et al., 2021a; Murga-Garrido et al., 2022). These changes are also linked with higher serum levels of tumor necrosis factor-α, also called TNF-α, and interleukin-6, also called IL-6. Together, these changes may form a cycle in which gut bacterial imbalance, metabolic problems, and inflammation affect each other (Yuan et al., 2021a; Burananat et al., 2025; Newman et al., 2023). Observational studies and Mendelian randomization studies also suggest that gut dysbiosis may help cause obesity, rather than only appearing after obesity has already developed (Li et al., 2024; Lu et al., 2024). The pubertal inflammatory-susceptibility framework is also relevant to metabolic remodeling. Pubertal obesity is closely associated with insulin resistance and abnormal sexual development, suggesting multisystem dysregulation beyond caloric excess (Huang and Roth, 2021; Qian et al., 2024). Hormonal changes during puberty may alter gut barrier integrity and increase vulnerability to microbial lipopolysaccharide (LPS) translocation, thereby promoting systemic inflammatory responses (Li et al., 2021). At the same time, obesity-related branched-chain amino acid (BCAA) accumulation should be interpreted as a multifactorial metabolic alteration influenced by dietary intake, hepatic and muscle metabolism, insulin resistance, and gut microbial BCAA transport or metabolism, rather than as a microbiota-driven change alone (Moran-Ramos et al., 2021; Yuan et al., 2023; Del Chierico et al., 2021). These mechanisms may jointly worsen insulin resistance, adipose tissue inflammation, and lipid storage during adolescence. These pubertal-specific vulnerabilities, when combined with dysbiosis-driven inflammation, also predispose affected individuals to comorbid conditions such as metabolic-associated fatty liver disease and metabolic syndrome (Rohani et al., 2025; Wierzbicka-Rucinska et al., 2025). Collectively, these findings support reconceptualizing childhood and adolescent obesity as a disorder rooted in disrupted gut microbiota–metabolism–immune crosstalk, superimposed upon a background of pubertal chronic inflammatory susceptibility (Fiore et al., 2023). This ecological perspective—shifting the focus from isolated energy balance to network-level dysregulation—offers a new framework for understanding the pathogenesis of pediatric obesity. Figure 1 shows the main differences between healthy conditions and obesity conditions in gut microbiota composition, intestinal barrier function, and immune-inflammatory status.

### 1.2 The gut microbiota as a signal converter that changes dietary substrates into bioactive metabolites

Childhood and adolescence are important periods for the development of gut microbiota, with lasting implications for metabolism and immune function (Carson et al., 2023; Jian et al., 2021). The gut microbiota functions as a metabolic converter, by transforming dietary components into small active molecules, especially SCFAs, bile acids, and indole derivatives (Zhou et al., 2023; Fiore et al., 2022; Koller et al., 2025). Diet directly modulates this process. High-energy, low-fiber dietary patterns reduce microbial diversity and disrupt gut microbial function. Diets rich in complex carbohydrates may help maintain a gut environment that supports better metabolism (Bagheri et al., 2022; Orbe-Orihuela et al., 2022). Thus, the types of gut bacteria microbial composition and metabolic capacity determine how dietary inputs are converted into host-sensed chemical signals (Arnoldini et al., 2025; Yu et al., 2025).

When this signaling pathway is disrupted, it plays a critical role in obesity among children and adolescents: some beneficial metabolic pathways are downregulated, while certain harmful pathways become overactive, collectively altering the overall metabolite profile (Del Chierico et al., 2021; Murga-Garrido et al., 2023; Squillario et al., 2023). These metabolites influence gut hormone release, immune cell activity, and whole-body energy balance. They may do this by interacting with receptors such as G protein-coupled receptor 41 (GPR41) and G protein-coupled receptor 43 (GPR43). They may also weaken the intestinal barrier, which can lead to long-term mild inflammation and metabolic problems (Koller et al., 2025; Klag et al., 2026; Li S. et al., 2025). Diet is one of the main factors that controls this process. Higher intake of ultra-processed foods is associated with reduced SCFA production and increased pro-inflammatory products. A high-fiber diet may support the production of anti-inflammatory metabolites (Calcaterra et al., 2023; Deligeorgopoulou et al., 2025; Ouradova et al., 2025). This metabolic function is not the same in every person or every population. Obesity-related gut microbiota patterns have also been reported in specific groups, including Chinese and Mexican cohorts (Murga-Garrido et al., 2022; Balakrishnan et al., 2021; Li et al., 2022). Childhood and adolescent obesity can therefore be understood as a problem linked to abnormal signals from the gut microbiota. Studying these disturbed functional patterns may help explain how diet, gut microbiota, and the host affect each other, and supports the development of more targeted intervention strategies (Zhang and Dang, 2022; de Cuevillas et al., 2022).

### 1.3 SCFAs and novel microbial metabolites that shape the “microbiota-immune-obesity” axis

The metabolites produced through this signaling process are important molecules in the microbiota–immune–obesity axis (Zhou et al., 2023; Jiang et al., 2025). SCFAs constitute the most studied effectors: they act as an energy source for enterocytes, thereby preserving barrier integrity; they activate GPR41/43 to facilitate secretion of peptide YY (PYY) and glucagon-like peptide-1 (GLP-1), governing appetite and insulin sensitivity; and they inhibit HDAC, modulating immune responses through epigenetic mechanisms (Fiore et al., 2022; Li S. et al., 2025). Outside the gut, SCFAs may also affect whole-body energy metabolism and body composition through the hypothalamic-pituitary-adrenal (HPA) axis and the gut–muscle axis (Liang et al., 2021; Visuthranukul et al., 2024a). In children and adolescents with obesity, total SCFA levels are often reduced, with concomitant shifts in the relative proportions of acetate, propionate, and butyrate (Deligeorgopoulou et al., 2025; Chan et al., 2026). This altered SCFA profile is associated with diminished metabolic protection (Gyarmati et al., 2021; Yu X. et al., 2023).

Beyond SCFAs, other microbial metabolites should be considered according to their biochemical origin and dominant host signaling pathways, including bile acid derivatives, aromatic amino acid catabolites, and endocannabinoid-like lipids. Secondary bile acids can act through FXR and TGR5 and may affect glucose metabolism, lipid metabolism, and energy balance (Chan et al., 2026; Giannini et al., 2022). AAA metabolites, such as tryptophan-derived indole-3-propionic acid, can activate the aryl hydrocarbon receptor (AHR). This process may help regulatory T-cell (Treg) differentiation and may directly affect immune balance in the gut and in the whole body (Jiang et al., 2025; Rivera et al., 2025). Other metabolites, including 5-aminovaleric acid and 1,4-methylimidazole acetic acid, have been demonstrated to target on pathways such as wingless-related integration site (Wnt)/β-catenin and Janus kinase/signal transducer and activator of transcription (JAK/STAT), participating in obesity and its complications (Deng et al., 2025; Zhao et al., 2025). Together, these metabolites establish an integrated network in which global balance dictates the stability of the microbiota–immune–obesity axis (Granato et al., 2025; Jardon et al., 2025; Wei et al., 2023).

This metabolite network may affect the body through several connected ways. Some helpful metabolites may promote regulatory T cell, also called Treg, differentiation and strengthen the intestinal barrier, thereby helping to reduce long-term mild inflammation (Jiang et al., 2025; Newman et al., 2023; Czarnowski et al., 2023). When the system is out of balance, intestinal permeability increases. This can allow endogenous antigens to move across the intestinal barrier, which may keep the immune system active and make metabolic problems worse (Al-Daghri et al., 2021; Lin X. et al., 2025). Metabolites may also bind directly to receptors on immune cells, affect barrier function, or change host enzyme activity and gene expression. These changes may help regulate fat storage (Zhou et al., 2023; Xia et al., 2025). This interaction may also be different at different stages of growth. Changes in the metabolite profile during puberty may have important long-term effects on health (Jian et al., 2021; Vander Wyst et al., 2021). Studying this network offers researchers a complementary approach to understand pediatric obesity and its complications, such as insulin resistance and fatty liver, while also informing more targeted nutrition strategies for different groups of children and adolescents with obesity (Fiore et al., 2023). The objective is to reestablish network equilibrium by reconstructing the microbiota and its metabolite output, thereby creating new opportunities for obesity prevention and treatment (Mo et al., 2025; Visuthranukul et al., 2024b; Wu et al., 2025).

## 2 Core regulatory mechanisms of SCFAs in childhood and adolescent obesity

### 2.1 Dietary fiber, age-related SCFA-producing bacteria, and context-dependent SCFA alterations in obesity

Dietary fiber is the main material used by gut bacteria to produce SCFAs. These SCFAs mainly include acetate, propionate, and butyrate, with an approximate ratio of 60:20:20. They are closely related to intestinal balance, immune development, and energy balance (Akagbosu et al., 2025). Different types of fiber, such as soluble fiber, resistant starch, and xylans, need to be fermented by anaerobic bacteria before they can become active metabolites (Akagbosu et al., 2022). A diet with enough fiber, such as a Mediterranean-style diet, can increase SCFA levels in the gut and reduce inflammatory markers. But a Western-style diet that is low in fiber, high in processed foods, and often linked with high salt intake may reduce butyrate production and weaken the mucus barrier in the colon. These changes may create conditions that make obesity more likely to develop (Akagbosu et al., 2025; Agustina et al., 2025).

However, the role of SCFAs in obesity should be interpreted cautiously. SCFAs have well-recognized beneficial effects, including serving as energy sources for colonocytes, strengthening epithelial barrier function, activating GPR41/GPR43-mediated gut hormone release, and regulating immune responses through GPCR signaling and HDAC inhibition (Fiore et al., 2022; Li S. et al., 2025; Pînzariu et al., 2025). At the same time, SCFAs are also fermentation products that may increase energy harvest from otherwise indigestible carbohydrates, and the efficiency of microbial fermentation varies substantially among individuals (Bäckhed et al., 2005; Arnoldini et al., 2025). Therefore, pediatric obesity should not be described as a universal state of SCFA deficiency. Current evidence is more consistent with context-dependent SCFA dysregulation, involving changes in SCFA-producing taxa, substrate availability, fecal and circulating SCFA concentrations, intestinal absorption, receptor sensitivity, and downstream immune-metabolic signaling (Li S. et al., 2025; Gyarmati et al., 2021; Pînzariu et al., 2025). This distinction is important because fecal SCFA levels do not directly reflect total SCFA production or host exposure, and human studies have reported heterogeneous or even contradictory associations between SCFAs and adiposity (Gyarmati et al., 2021; Nguyen Tran et al., 2025; Jaimes et al., 2021).

The SCFA-producing microbiota in children and adolescents changes clearly with age. In the first 2 years of life, birth mode and feeding method help shape the early gut bacterial community. Infants born vaginally and fed with breast milk often show higher levels of *Lactobacillus* and *Bifidobacterium*. By contrast, infants born by cesarean delivery may have more skin-related staphylococci and fewer SCFA-producing bacteria (Agustina et al., 2025). As children begin to eat more types of food, strictly anaerobic bacteria that produce SCFAs, such as *Faecalibacterium prausnitzii, Roseburia*, and *Eubacterium*, can become more stable in the gut and gradually become more common (McCann et al., 2021; Vázquez-Bolea et al., 2025). During puberty, changes in sex hormones and eating habits may further reshape the gut microbiota. *Clostridium* clusters IV and XIVa, Fibrobacteraceae, and Ruminococcaceae may become important SCFA-producing bacteria with high activity. At this stage, the gut microbiota slowly becomes more similar to that of adults, but it can still be strongly affected by diet and environmental factors (Carson et al., 2023; Akagbosu et al., 2025, 2022).

Obesity may disrupt this normal developmental trajectory, but the microbial changes are not uniform across cohorts. Compared with normal-weight peers, obese children and adolescents often show fewer beneficial SCFA-producing bacteria, such as *Faecalibacterium prausnitzii* and *Bifidobacterium*. They also show an uneven Bacteroidetes/Firmicutes ratio, which may reduce the production of acetate, propionate, and butyrate (Yuan et al., 2021a; Li S. et al., 2025; Ahmed et al., 2021). Nevertheless, phylum-level markers such as the Firmicutes/Bacteroidetes (F/B) ratio should not be used as a reliable standalone indicator of obesity. The F/B ratio is a coarse taxonomic index that does not capture strain-level differences, functional gene capacity, SCFA production, bile acid metabolism, or immune-modulatory potential. Its association with obesity has been inconsistent across pediatric and adult cohorts, and it may be strongly influenced by diet, age, ethnicity, geography, and analytical methods (Gyarmati et al., 2021; McCann et al., 2021; Vázquez-Bolea et al., 2025). Therefore, obesity-associated microbiome changes should be described in terms of specific taxa, microbial functions, metabolite profiles, and host immune-metabolic context rather than relying on the F/B ratio alone.

The heterogeneity of human studies likely reflects multiple confounding factors. Dietary fiber intake, ultra-processed food consumption, sugar-sweetened beverages, physical activity, sleep, antibiotic exposure, anti-obesity or antidiabetic medications, age, pubertal stage, ethnicity, geography, early-life feeding, and comorbidities can all influence gut microbiota composition and SCFA metabolism (Calcaterra et al., 2025; Morgado et al., 2023; Glaros et al., 2025). Study design and methodology also contribute to inconsistent findings, including cross-sectional vs. longitudinal designs, sample size, sequencing platform, 16S rRNA gene sequencing vs. shotgun metagenomics, DNA extraction procedures, bioinformatic pipelines, and whether SCFAs are measured in feces, serum, or other biological compartments (McCann et al., 2021; Zöggeler et al., 2025; Gawlik et al., 2021). Future studies should therefore integrate dietary records, lifestyle data, medication history, pubertal status, ethnicity, multi-omics profiling, and standardized SCFA measurement to clarify whether specific SCFA-related changes are causal, compensatory, or secondary to obesity.

The resulting SCFA insufficiency impairs gut hormone secretion and thus disrupts appetite control and energy balance; through butyrate insufficiency, it also compromises epithelial barrier integrity, induces endotoxemia, and elicits chronic low-grade inflammation that promotes insulin resistance (Xia et al., 2025; Ayala-García et al., 2024). Because of this, restoring the ability to produce SCFAs may be useful in obese children. Prebiotics, such as inulin, or targeted probiotics can increase beneficial bacteria and fecal SCFA levels. These changes may lead to better metabolic outcomes (Visuthranukul et al., 2024b; Andriyas et al., 2025; Kilic Yildirim et al., 2023). A comparison of the contrasting microbiota and metabolic features across developmental stages and in obesity is presented in Table 1.

**Table 1:** Gut microbiota and metabolic profile characteristics at different developmental stages and in obese states in children and adolescents.

Developmental status	Key microbial abundance changes	Major metabolites	Immune response characteristics	References
Early life (0–2 years)	Vaginally delivered infants: *Lactobacillus* dominates; cesarean-delivered infants: *Bifidobacterium* and Lactobacillus are enriched; *Staphylococcus* dominates cesarean-delivered infants.	Mainly acetate and lactate, dynamically changing with the feeding pattern.	The microbiota drives immune system maturation and establishes oral tolerance.	Agustina et al., 2025; Oksanen et al., 2025
Toddler to preschool years	Increased microbial diversity; SCFA-producing bacteria, including *Faecalibacterium*, Roseburia, and *Eubacterium* begin to colonize.	Gradual increase and stabilization of SCFA production.	As the function of Treg cells and other immune components matures, the immune system and microbiota develop cooperatively.	McCann et al., 2021; Vázquez-Bolea et al., 2025
Adolescence	*Clostridium* clusters IV and XIVa, *Lachnospiraceae, and Ruminococcaceae* become core SCFA-producing microbiota.	High and stable SCFA production; elevated proportion of secondary bile acids and other metabolites.	Sex hormones and microbiota metabolites jointly influence immunity, increasing susceptibility to metabolic dysregulation.	Carson et al., 2023; Jian et al., 2021; Akagbosu et al., 2025, 2022
Obese status	Decreased microbial diversity; reduced beneficial bacteria, including *Faecalibacterium, Akkermansia*, and *Bifidobacterium*; altered microbial composition and functional capacity; the *Firmicutes/Bacteroidetes* ratio is inconsistent and should not be used as a standalone obesity marker.	Altered SCFA profiles with inconsistent direction across studies; reduced 4-HPAA in some cohorts; BCAA accumulation influenced by diet, insulin resistance, hepatic and muscle metabolism, and microbiota; abnormal bile acid profile.	Increased LPS translocation, elevated TNF-α, IL-6, impaired Treg function, and chronic low-grade inflammation.	Zhang and Dang, 2022; Yuan et al., 2021a; Burananat et al., 2025; Newman et al., 2023; Yuan et al., 2021b

### 2.2 SCFAs in the regulation of local gut immunity

SCFAs are key signals maintaining intestinal immune homeostasis in children and adolescents, acting through GPCRs and HDAC inhibition (Jiang et al., 2025; Akagbosu et al., 2022; Agustina et al., 2025).

Within the GPCR axis, GPR43 is broadly expressed on lymphoid tissues and immune cells. SCFAs binding to GPR43 dampens pro-inflammatory cascades such as the nuclear factor kappa B (NF-κB) pathway, reducing TNF-α and IL-6 secretion while promoting Treg differentiation (Newman et al., 2023). G-protein-coupled receptor 109A (GPR109a), mainly activated by butyrate and highly expressed on intestinal macrophages, drives apoptosis of inflammatory cells and upregulates the anti-inflammatory cytokine interleukin-10 (IL-10; Akagbosu et al., 2022; Agustina et al., 2025). In the HDAC arm, SCFAs enter the nucleus, inhibit HDAC activity, and induce histone hyperacetylation, which activates the transcription of anti-inflammatory cytokines and silences pro-inflammatory genes, further enforcing an anti-inflammatory phenotype (Zhang and Dang, 2022; Fiore et al., 2022). At the cellular level, these pathways work together to push macrophages toward the anti-inflammatory M2 phenotype and limit M1 polarization. This effect is partly related to macrophage metabolic reprogramming caused by HDAC inhibition. Although such mechanisms have been linked to improved insulin sensitivity and reduced hepatic lipid accumulation in experimental models, direct clinical evidence in children remains limited (Newman et al., 2023; Del Chierico et al., 2021; Czarnowski et al., 2023). SCFAs also control excessive neutrophil activation through GPR43, including chemotaxis, reactive oxygen species production, and apoptosis. This helps reduce intestinal tissue damage and keeps the epithelial barrier intact (Zhou et al., 2023; Deligeorgopoulou et al., 2025; Pînzariu et al., 2025).

In pediatric obesity, gut dysbiosis can lower SCFA production and weaken these immune-regulating brakes. When SCFA-related control of NF-κB suppression and HDAC-driven transcriptional regulation becomes weaker, TNF-α and IL-6 production can increase. At the same time, weaker control of macrophage M1/M2 polarization and continued neutrophil activation can worsen tissue damage and compromise the epithelial barrier (Fiore et al., 2022; Li S. et al., 2025; Pînzariu et al., 2025). Overall, impaired SCFA-mediated local immune regulation may contribute to epithelial barrier dysfunction and chronic low-grade intestinal inflammation, which can interact with systemic metabolic abnormalities (Newman et al., 2023; Li et al., 2024).

SCFAs are also important for building and maintaining the intestinal epithelial barrier. Butyrate serves as the main energy source for colonocytes. It supports cell proliferation, cell differentiation, and mucosal homeostasis (Pînzariu et al., 2025). SCFAs also suppress HDACs to enhance the expression of tight-junction genes and interact with GPR43/GPR109a to facilitate barrier protein synthesis through the mitogen-activated protein kinase (MAPK)/phosphatidylinositol 3-kinase (PI3K)-Akt pathway; acetate and butyrate, for example, significantly elevate zonula occludens-1 (ZO-1) and occludin levels, strengthening the physical barrier (Fiore et al., 2022; Akagbosu et al., 2022; Pi et al., 2024). Additionally, SCFAs help stabilize hypoxia-inducible factor, stimulate intestinal trefoil factor to speed up mucosal repair, and attenuate NOD-like receptor protein 3 (NLRP3) inflammasome activation (Akagbosu et al., 2025; Agustina et al., 2025).

**Figure 2:**
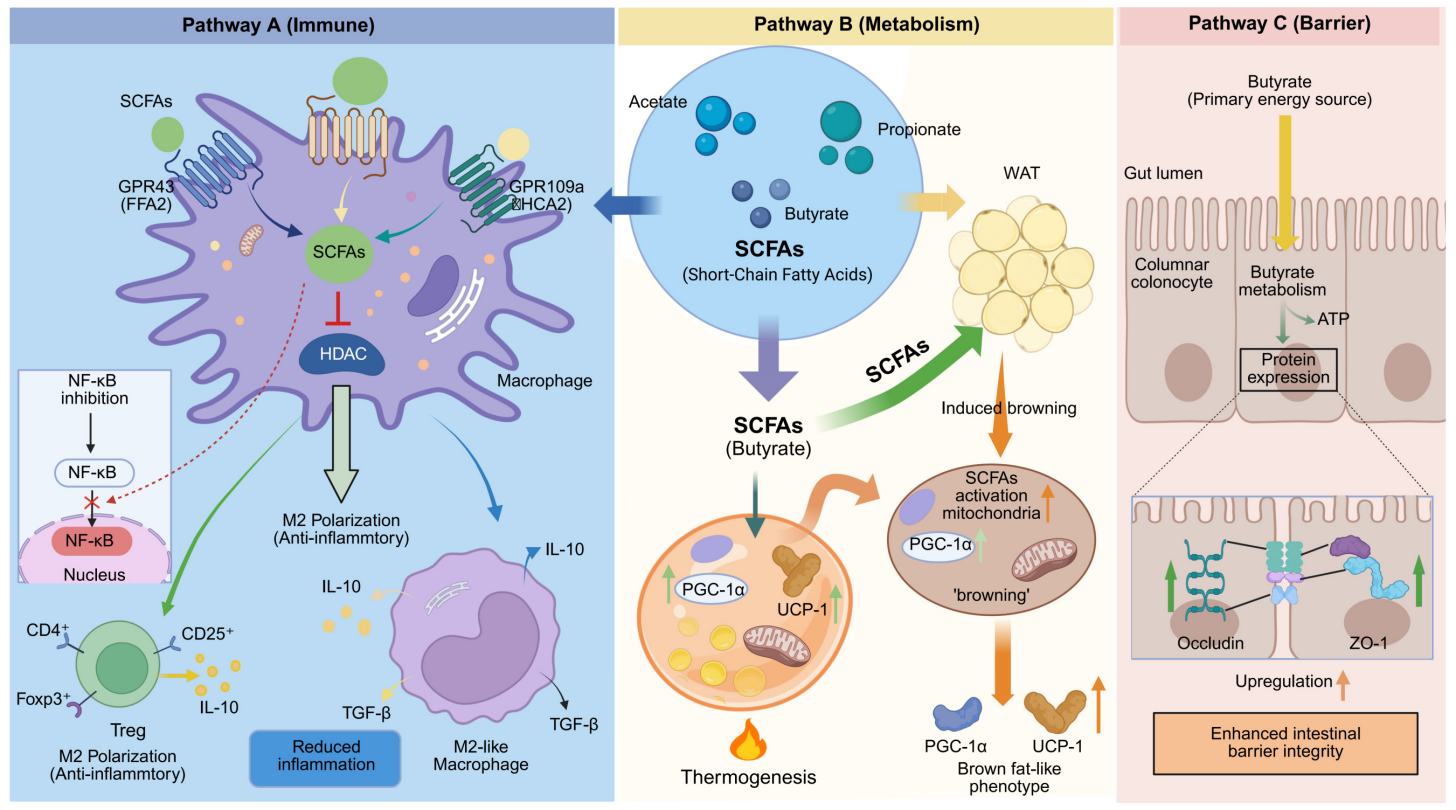
Integrated molecular mechanisms mediated by SCFAs in the gut–immune–metabolic axis of childhood and adolescent obesity. Created in BioRender. Qy, Z. (2026). https://BioRender.com/qpv2f15.

In obesity, reduced SCFA production can leave the epithelium with less energy and make tight junctions looser. This raises intestinal permeability and makes it easier for LPS to move across the gut barrier, a process known as metabolic endotoxemia (Zhang and Dang, 2022). LPS then activates Toll-like receptor 4 (TLR4) and NF-κB pathways, inducing TNF-α and IL-6 release and triggering systemic chronic low-grade inflammation that connects gut dysbiosis to insulin resistance and fat accumulation (Fiore et al., 2022; Akagbosu et al., 2025; Ahmed et al., 2021). However, evidence that SCFA-targeted interventions directly prevent long-term metabolic sequelae in children remains limited. Current pediatric data mainly support the potential of fiber or prebiotic strategies to reshape the microbiota–metabolite environment and improve selected metabolic indices, rather than proving direct disease-modifying effects of SCFAs themselves (Mo et al., 2025; Visuthranukul et al., 2024b). Figure 2 outlines the multi-functional effects of SCFAs on immune cell function, adipose tissue thermogenesis, and gut barrier integrity.

### 2.3 Integrated regulation of systemic immunity and metabolism by SCFAs

During childhood and adolescence, butyrate and propionate are absorbed through the portal vein and help form a gut–systemic immune regulatory axis. Experimental studies suggest that SCFAs may influence immune cell differentiation through HDAC inhibition and GPCR-related signaling, including effects on Treg differentiation and myeloid cell polarization (Jiang et al., 2025; Newman et al., 2023; Akagbosu et al., 2025). However, the concept of SCFA-mediated “immune training” in humans, especially in children and adolescents, remains largely inferential and should be interpreted cautiously. Immune cells influenced by SCFA-related signaling may also affect peripheral tissues, including adipose tissue. In experimental models, SCFAs have been associated with reduced M1 macrophage polarization, increased M2-like macrophage features, and enhanced Treg-related anti-inflammatory responses in adipose tissue (Li et al., 2024; Akagbosu et al., 2025; Ahmed et al., 2021). In humans, however, direct evidence that SCFAs induce durable immune training and thereby remodel adipose tissue immunity during pediatric obesity remains limited. These anti-inflammatory effects may reduce the release of TNF-α and IL-6 and increase anti-inflammatory mediators such as IL-10 and adiponectin, but the extent to which this pathway operates in children with obesity requires further validation.

**Figure 3:**
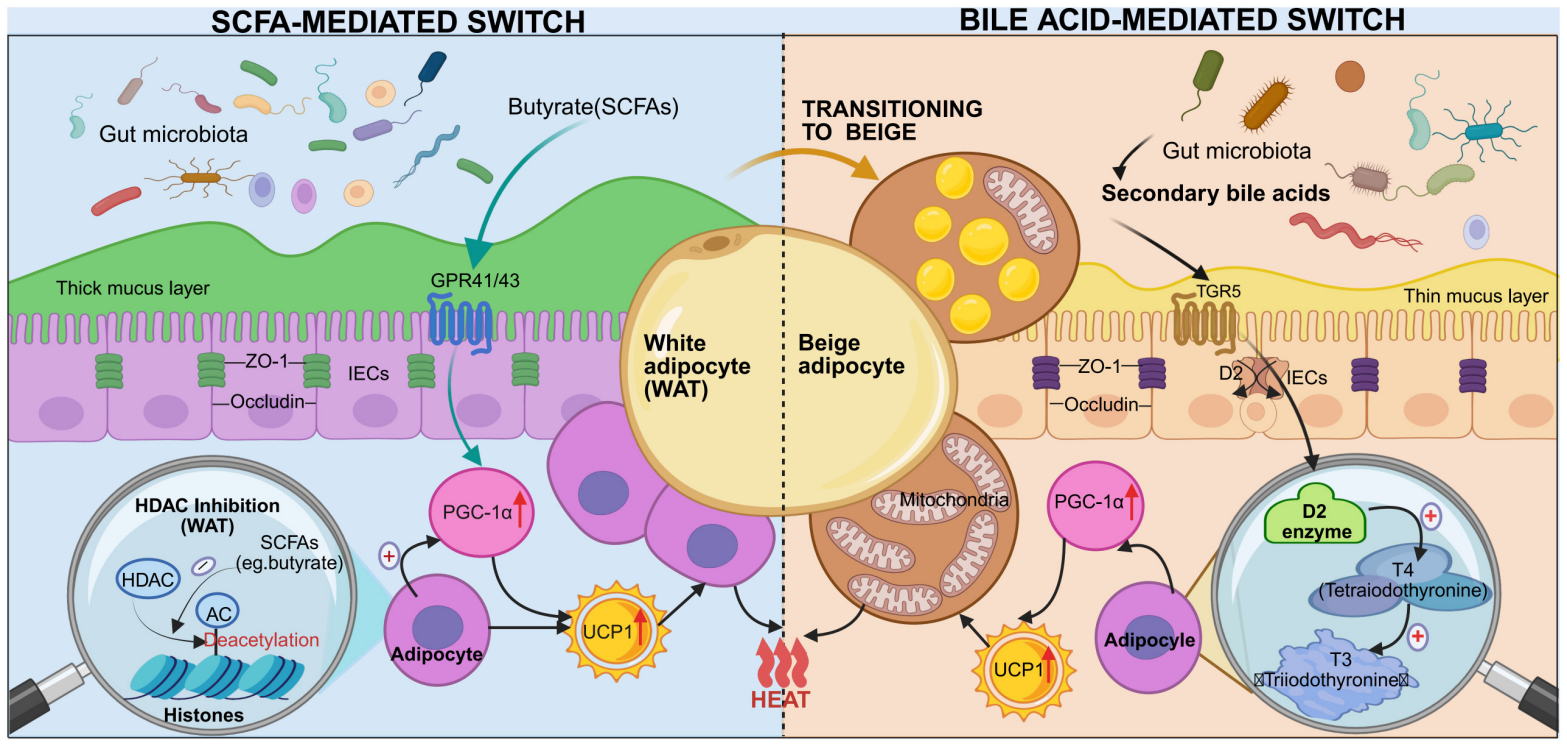
Gut–adipose axis metabolic switch mediated by microbial metabolites. Created in BioRender. Xing, Z. (2026). https://BioRender.com/z413ny2.

SCFAs also act as vital signaling molecules within the gut–adipose axis, orchestrating comprehensive modulation of adipose tissue energy metabolism and phenotypic remodeling. On one hand, they activate GPR41/43 on adipocytes, suppressing transcription of sterol regulatory element-binding protein 1c (SREBP-1c) and increasing the activity of lipolytic enzymes such as lipoprotein lipase, thereby restricting triglyceride accumulation in white adipose tissue (WAT). Meanwhile, they improve the immune milieu by blocking pro-inflammatory activation of macrophages (Newman et al., 2023; Al-Daghri et al., 2021; Akagbosu et al., 2022). On the other hand, experimental studies also suggest that SCFAs may regulate adipose tissue thermogenic programs by increasing the expression of thermogenic genes such as uncoupling protein 1 (UCP1) and peroxisome proliferator-activated receptor gamma coactivator 1-alpha (PGC-1α) and by engaging pathways related to peroxisome proliferator-activated receptor gamma (PPARγ)/PGC-1α and β3-adrenergic receptor (β3-AR)/p38 mitogen-activated protein kinase (p38 MAPK) signaling (Al-Daghri et al., 2021; Alcázar et al., 2022). However, whether SCFAs directly drive beige adipocyte generation through this pathway in humans remains uncertain, and pediatric evidence is particularly limited. SCFAs also strengthen the adipose thermogenic program by activating gut–brain sympathetic circuits (Verduci et al., 2021b). Since children and adolescents have relatively more BAT than adults, SCFA-mediated thermogenesis may be especially important for weight regulation during puberty, although this remains largely extrapolated from animal data (Alcázar et al., 2022). In obesity, gut dysbiosis alters SCFA profiles and contribute to impaired adipose tissue metabolic regulation, but the extent to which this pathway directly causes adipose tissue-specific insulin resistance in humans requires further validation (Jardon et al., 2025). Although reduced BAT activity is bidirectionally linked to obesity-related complications such as non-alcoholic fatty liver disease (NAFLD; Ahmed et al., 2021). Despite these promising preclinical findings, SCFAs have attracted considerable translational interest as a potential “metabolic switch” that activates BAT and induces WAT browning (Verduci et al., 2021b). Accordingly, dietary strategies such as fiber and prebiotics that remodel the microbiota metabolite landscape and optimize SCFA production and composition may help improve SCFA-related metabolic signaling, offering novel targets for preventing and treating obesity and metabolic syndrome in the young (Al-Daghri et al., 2021; Alcázar et al., 2022). The biological effects exerted by SCFAs through distinct receptors and pathways, together with their functional impairments in obesity, are summarized in Figure 3 and Table 2.

**Table 2:** Evidence-aware summary of SCFA-related immune-metabolic pathways in pediatric obesity.

Regulatory dimension/tissue system	Obesity-related alteration	Core receptor targets	Functional interpretation	Evidence basis and translational limitation	References
Gut local immunity	Altered SCFA availability or signaling may weaken NF-κB suppression and HDAC-associated anti-inflammatory regulation; macrophage M1/M2 balance, Treg responses, and neutrophil activation may be affected.	GPR43, GPR109a, HDAC; NF-κB, IL-10-related signaling, and histone acetylation.	Supports local immune homeostasis and may limit excessive TNF-α/IL-6 production and epithelial inflammatory injury.	Mechanistic evidence is strong in cell and animal studies. Pediatric human evidence mainly supports associations between SCFA-related dysbiosis and inflammatory phenotypes, not definitive causality.	Newman et al., 2023; Li et al., 2024; Del Chierico et al., 2021; Fiore et al., 2022; Czarnowski et al., 2023; Akagbosu et al., 2022; Agustina et al., 2025; Pînzariu et al., 2025
Gut epithelial barrier integrity	Reduced butyrate availability or altered SCFA signaling may compromise colonocyte energy supply, tight-junction support, and mucosal repair.	GPR41, GPR43, GPR109a, HDAC; MAPK/PI3K-Akt; HIF and trefoil factor-related pathways; autophagy-related stress responses and NLRP3 inflammasome modulation.	May increase intestinal permeability and facilitate LPS translocation, contributing to metabolic endotoxemia.	Barrier effects are supported by experimental and mechanistic evidence. Direct pediatric clinical evidence that SCFA-targeted interventions prevent long-term metabolic complications remains limited.	Zhang and Dang, 2022; Fiore et al., 2022; Akagbosu et al., 2025, 2022; Pînzariu et al., 2025; Pi et al., 2024
Developmental immune training	SCFA-related signals may influence developing immune-cell differentiation, but durable “immune training” in humans should not be assumed.	HDAC inhibition and GPCR signaling affecting Treg differentiation, myeloid cell polarization, and inflammatory gene transcription.	May contribute to immune tolerance and lower inflammatory tone. This should be described as immune modulation rather than confirmed immune training in pediatric humans.	Mainly experimental and mechanistic evidence. Direct evidence for long-lasting SCFA-mediated immune training in children and adolescents is limited.	Jiang et al., 2025; Carson et al., 2023; Newman et al., 2023; Arnoldini et al., 2025; Akagbosu et al., 2025
Adipose tissue metabolism	Altered SCFA signaling may influence adipocyte lipid handling and thermogenic programs, but direct beige adipocyte generation in humans is uncertain.	GPR41, GPR43, HDAC; SREBP-1c, lipolytic enzymes, UCP1, PGC-1α, PPARγ/PGC-1α, and β3-AR/p38 MAPK-related signaling.	Potential effects include reduced triglyceride accumulation and increased thermogenic gene expression in experimental models. Human BAT activation and WAT browning remain less certain.	Evidence for β3-AR/p38 MAPK-mediated beige adipocyte generation comes mainly from cell and animal models. Pediatric validation is particularly limited.	Jardon et al., 2025; Al-Daghri et al., 2021; Akagbosu et al., 2022; Ahmed et al., 2021; Alcázar et al., 2022; Verduci et al., 2021b
Gut-brain axis regulation	Altered SCFA profiles may reduce gut hormone responses and contribute to appetite dysregulation, but responses vary according to diet, baseline microbiota, and host metabolism.	GPR41/GPR43-mediated PYY and GLP-1 secretion; vagal and hypothalamic appetite pathways.	May influence satiety signaling and energy intake as part of the gut-brain axis.	Supported by mechanistic and human association/intervention evidence to varying degrees. Direct pediatric clinical efficacy remains context-dependent.	Yu et al., 2025; Klag et al., 2026; Liang et al., 2021; Visuthranukul et al., 2024a; Panichsillaphakit et al., 2025
Integrated systemic metabolic outcomes	SCFA-related barrier, immune, adipose, and gut-brain pathways may interact with insulin resistance, hepatic lipid accumulation, and metabolic syndrome.	Combined GPCR/HDAC signaling; endotoxemia-TLR4/NF-κB axis; adipose inflammatory signaling; gut hormone pathways.	Provides an integrated mechanistic framework linking gut microbial metabolites with systemic metabolism.	Should be presented as biologically plausible and partially supported by experimental and observational evidence, not as a clinically established SCFA-driven therapeutic mechanism in children.	Zhang and Dang, 2022; Newman et al., 2023; Li et al., 2024; Jardon et al., 2025; Al-Daghri et al., 2021; Akagbosu et al., 2025; Pînzariu et al., 2025; Ahmed et al., 2021; Alcázar et al., 2022; Verduci et al., 2021b

## 3 Unique mechanisms of novel microbial metabolites in childhood and adolescent obesity

Beyond SCFAs, the gut microbiota generates a broad spectrum of structurally distinct bioactive compounds from host-derived and dietary substrates, predominantly aromatic amino acid (AAA) metabolites, secondary bile acids and N-acyl amides. These molecules help regulate energy balance, immune balance, and glucose and lipid metabolism through direct or indirect signaling pathways. This provides new understanding of how childhood and adolescent obesity develops (Wei et al., 2023; Jaimes et al., 2021; Andriyas et al., 2025).

### 3.1 AAA metabolites as emerging therapeutic candidates

4-HPAA, a major metabolite formed from phenylalanine, is produced by gut microbiota through AAA fermentation and flavonol metabolism. Its production efficiency depends on dietary flavonoid consumption and the abundance of *Flavonifractor plautii* (Ahmed et al., 2021). Among children and adolescents, obese individuals show significantly lower 4-HPAA levels in feces and serum, and this metabolite has a negative relationship with adiposity (Wei et al., 2023; Li Q. R. et al., 2025). Supplementation with 4-HPAA suppresses weight gain and fat accumulation without obvious adverse effects (Álvarez-Arraño and Martín-Peláez, 2021). Mechanistically, 4-HPAA signals through GPR43 and the PI3K/Akt pathway to enhance type 3 innate lymphoid cell (ILC3) proliferation and interleukin-22 (IL-22) secretion. This process promotes the production of antimicrobial peptides, strengthens the intestinal mucus barrier, and reduces adipose tissue inflammation induced by bacterial endotoxin translocation (Jiang et al., 2025).

Metabolically, 4-HPAA works through several pathways. It limits fatty acid uptake by downregulating the expression of cluster of differentiation 36 (CD36), Niemann-Pick C1-like 1 (NPC1L1), and fatty acid-binding protein (FABP) in intestinal epithelial cells. This effect is linked to two processes. 4-HPAA suppresses PPARα activity, which inhibits CD36 transcription. It also activates AMP-activated protein kinase (AMPK), which promotes the phosphorylation-dependent degradation of NPC1L1 (Jiang et al., 2025; Álvarez-Arraño and Martín-Peláez, 2021; Yin et al., 2025). In the liver, 4-HPAA activates AMPKα, increases fatty acid oxidation, and reduces *de novo* lipogenesis. Through this process, it can reverse hepatic steatosis in adolescents (Ahmed et al., 2021). After 4-HPAA intervention, obese children show significantly lower serum free fatty acid and total cholesterol levels. This outcome is directly related to reduced intestinal fatty acid absorption (Li Q. R. et al., 2025). These findings have supported the development of dietary strategies that increase 4-HPAA-producing gut microbiota. Clinical evidence shows that these strategies can raise beneficial metabolite levels and improve metabolic parameters in obese adolescents (Visuthranukul et al., 2024b; Andriyas et al., 2025). Still, data on pediatric safety and efficacy are limited, and large randomized controlled trials (RCTs) are needed to confirm its long-term therapeutic effects (Fahim et al., 2025).

Indole derivatives—including indole-3-acetic acid (IAA) and indole-3-carbaldehyde (IAld), are generated via microbial tryptophan metabolism, and are linked to microbial genera such as *Bifidobacterium* and *Lactobacillus* (Aragón-Vela et al., 2021). Their intestinal concentrations may change with gut microbiota maturation and pubertal development, while selected indole derivatives have been reported to be reduced or altered under obese conditions. Experimental studies suggest that these pathways may support intestinal stem cell activity, enhance tight-junction protein expression, suppress excessive NLRP3 inflammasome activation and pro-inflammatory cytokine release, reduce intestinal permeability, and promote IL-22-related mucosal immune responses (Deng et al., 2025; Prodam et al., 2025; Puértolas-Balint et al., 2025). Systemically, experimental evidence suggests that indole-related pathways may influence fatty acid uptake and oxidation in CD4? T cells, limit pro-inflammatory T helper 1 (Th1)/T helper 17 (Th17)-related responses, and support Treg-related immune regulation. However, direct evidence that indole derivatives increase Treg production in children and adolescents with obesity remains limited (Jiang et al., 2025; Sun D. et al., 2025). Metabolomic studies show that lower indole-3-propionic acid (IPA) levels in obese children are associated with systemic low-grade inflammation (Wei et al., 2023; Luo et al., 2025). Although indole derivatives represent promising candidates for microbiota–immune modulation, their therapeutic relevance, safety, and long-term efficacy in children and adolescents with obesity require further validation (Rivera et al., 2025).

### 3.2 Secondary bile acids and FXR/TGR5-related modulation of energy, glucose, and lipid metabolism

Secondary bile acids, mainly lithocholic acid and deoxycholic acid, are produced by gut bacteria, such as *Bacteroides* and *Clostridium*, from primary bile acids synthesized by the liver (Aragón-Vela et al., 2021). Their composition changes with age. Primary bile acids are dominant in the neonatal period. The proportion of secondary bile acids then gradually increases as the microbiota matures after school age, and a stable bile acid pool is fully formed by adolescence (Andriyas et al., 2025). In childhood obesity, disruption of the microbiota–bile acid axis plays an important role in metabolic disorders. Multi-omics studies show that abnormal secondary bile acid levels are closely linked to insulin resistance and dyslipidemia. Meta-analyses and systematic reviews have demonstrated significant associations between lower microbial diversity, impaired secondary bile acid synthesis, and pediatric obesity; however, these observational findings cannot establish causality, and causal inference requires validation through prospective cohort studies and Mendelian randomization approaches. Some obese children also have higher serum levels and abnormal composition of secondary bile acids. This abnormality is directly related to complications, such as NAFLD and insulin resistance (Wei et al., 2023; Zöggeler et al., 2025; Aqeel et al., 2025).

Secondary bile acids regulate energy metabolism through two receptors, FXR and TGR5. FXR activation increases the expression of fatty acid transporters, which promotes fatty acid uptake. It also stimulates fibroblast growth factor 19 (FGF19), which feeds back to inhibit hepatic cholesterol 7α-hydroxylase (CYP7A1) and helps maintain bile acid homeostasis. At the same time, FXR activation directly promotes hepatic gluconeogenesis through upregulation of phosphoenolpyruvate carboxykinase (PEPCK) and glucose-6-phosphatase (G6Pase), which tends to elevate blood glucose; however, the net effect on systemic insulin sensitivity is more complex and may be influenced by FGF19-mediated metabolic reprogramming and the tissue-specific context of FXR signaling (Giannini et al., 2022; Czarnowski et al., 2023; Aragón-Vela et al., 2021). TGR5 works through the AMPK pathway. In intestinal L cells, it promotes GLP-1 secretion, which helps reduce appetite and improve glucose regulation. In brown adipose tissue and skeletal muscle, it activates type 2 iodothyronine deiodinase. This enzyme converts thyroxine to triiodothyronine and raises UCP1 expression, which increases thermogenesis (Jiang et al., 2025; Giannini et al., 2022). TGR5 also reduces the release of pro-inflammatory cytokines from macrophages and helps relieve adipose tissue inflammation (Andriyas et al., 2025). The bile acid receptor agonist obeticholic acid, which acts through FXR/TGR5, is being explored as a potential treatment option for pediatric obesity. However, its safety and clinical efficacy still need further confirmation (Giannini et al., 2022).

### 3.3 N-acyl amides: regulation of appetite and inflammation via G protein-coupled receptors

Structurally similar to the body's own endocannabinoids, gut microbiota-derived N-acyl amides work as lipid signaling molecules. They help regulate energy balance and immune metabolism in children and adolescents by binding to GPCRs and classical cannabinoid receptors 1/2 (CB1/CB2; Zhou et al., 2023; Jiang et al., 2025; Wei et al., 2023). Their production is closely related to dietary fat and carbohydrate intake. Under obese conditions, dysbiosis can lead to abnormal accumulation of N-acyl amides, which is associated with adolescent hyperphagia and fat deposition (Wei et al., 2023; Andriyas et al., 2025).

In appetite regulation, N-acyl amides have two different effects through the gut–brain axis. They activate G protein-coupled receptor 119 (GPR119) on intestinal enteroendocrine cells and stimulate the secretion of satiety hormones, including GLP-1 and PYY. This helps limit food intake (Liang et al., 2021; Cheng et al., 2024). At the same time, certain subtypes bind to hypothalamic CB1, increase appetite drive, and form a vicious cycle of dysbiosis, metabolite abnormality, and energy accumulation. They also reduce lipolysis and promote lipid deposition through G-protein-coupled receptor 10 (GPR10; Wei et al., 2023; Al-Daghri et al., 2021; Aragón-Vela et al., 2021).

Beyond appetite, N-acyl amides also affect metabolic and immune balance through other pathways. In adipose tissue, they activate the PPARα pathway, which promotes fatty acid oxidation and improves insulin sensitivity (Cao et al., 2025). Within the intestinal immune microenvironment, engagement of CB2, G-protein-coupled receptor 55 (GPR55), and G-protein-coupled receptor 18 (GPR18) on immune cells modulates the release of pro-inflammatory cytokines such as TNF-α and IL-6. This helps reduce obesity-related chronic low-grade inflammation (Newman et al., 2023; Li et al., 2024; Cheng et al., 2024). Notably, the gut commensal *Faecalibacterium prausnitzii* expresses fatty acid amide hydrolase (FAAH), which carefully regulates local N-acyl ethanolamine levels and keeps a dynamic balance between immune and energy sensing (Klag et al., 2026). Given that the endocannabinoid system is not fully developed in children and adolescents, abnormal N-acyl amide accumulation can further destabilize metabolic regulation. Antagonists or inhibitors targeting GPR119 and CB1 represent promising strategies for precision intervention. Even so, their safety and long-term efficacy in the young require clinical assessment (Al-Daghri et al., 2021; Aragón-Vela et al., 2021).

**Figure 4:**
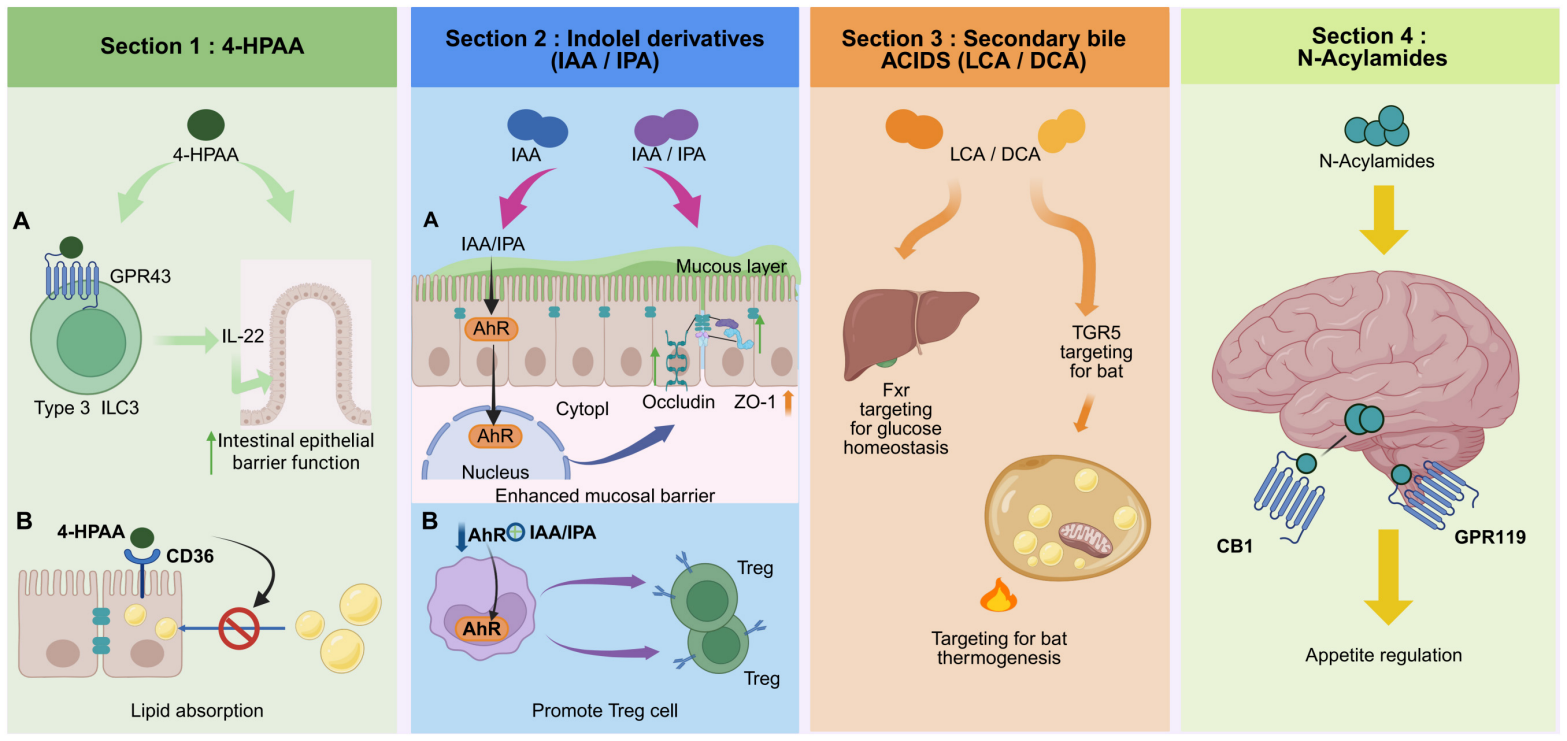
Comparative mechanisms of representative microbial metabolites involved in obesity regulation. Created in BioRender. Qy, Z. (2026). https://BioRender.com/cmch1aw.

The unique pathways through which these novel microbial metabolites participate in the microbiota–immune–obesity dialogue are summarized in Table 3. Evidence categories were used to distinguish experimentally validated mechanisms, pediatric observational associations, randomized or non-randomized clinical intervention evidence, and emerging hypotheses lacking direct pediatric therapeutic validation. Changes in the abundance and metabolic contributions of key functional bacterial groups are shown in Table 4, and the main pathways and targets of different microbial metabolite classes in childhood and adolescent obesity are illustrated in Figure 4.

**Table 3:** The unique pathways of novel microbial metabolites in the “microbiota-immune-obesity” dialogue.

Metabolite category	Representative molecule	Mainly produced by bacterial genera	Abnormal under obese conditions	Core receptor target	Regulatory effect on obesity	Evidence type and validation status	References
AAAs	4-HPAA	*Flavonifractor plautii*	Fecal and serum 4-HPAA concentrations *markedly reduced*.	Likely through GPR43, activating PI3K/Akt.	Activating ILC3s boosts IL-22 secretion and strengthens the gut barrier; downregulating intestinal fat-absorption proteins cuts fat accumulation.	Experimental mechanistic evidence plus pediatric association. The anti-obesity mechanism has been experimentally validated, but direct pediatric supplementation or receptor-targeted intervention has not been confirmed by randomized controlled trials. Therefore, 4-HPAA should be described as a mechanistically supported candidate rather than a clinically validated therapeutic target.	Jiang et al., 2025; Ahmed et al., 2021; Li Q. R. et al., 2025; Álvarez-Arraño and Martín-Peláez, 2021; Yin et al., 2025
Indole derivatives	IPA, IAA	*Bifidobacteria, Lactobacilli, etc*.	Production sharply drops, tightly linked to systemic low-grade inflammation.	Aromatic hydrocarbon receptor.	AHR-related signaling may support gut barrier integrity, limit excessive NLRP3 inflammasome activation, and influence Treg/Th1/Th17 immune balance; direct pediatric therapeutic validation remains limited.	Experimental mechanistic evidence plus human metabolomic association. Pediatric observational and metabolomic studies support associations with inflammation and cardiometabolic risk, but interventional evidence in children and adolescents remains insufficient.	Rivera et al., 2025; Deng et al., 2025; Andriyas et al., 2025; Sun D. et al., 2025; Luo et al., 2025
Secondary bile acid	Lithocholic acid, deoxycholic acid	*Bacteroides, Clostridium*	An imbalance in the bile acid pool composition is associated with MASLD and the progression of insulin resistance.	FXR, TGR5.	FXR activation regulates gluconeogenesis and lipid synthesis; TGR5 activation promotes GLP-1 secretion, enhances brown adipose tissue thermogenesis, and curbs macrophage inflammation.	Pediatric observational and multi-omics association plus established receptor biology. FXR/TGR5 signaling is biologically plausible and mechanistically supported, but bile acid receptor agonists should be regarded as translational candidates because pediatric obesity-specific safety and efficacy data remain limited.	Chan et al., 2026; Giannini et al., 2022; Wei et al., 2023; Zöggeler et al., 2025; Aragón-Vela et al., 2021; Aqeel et al., 2025; Mancera-Hurtado et al., 2023
N-Acylamide	N-Acylethanolamine	Various symbiotic bacteria, with *Faecalibacterium prausnitzii* modulating its levels.	Abnormal accumulation of metabolic profiles is positively correlated with increased appetite and fat accumulation during puberty.	CB1, CB2, GPR119, GPR55, GPR18, PPARα.	GPR119 triggers GLP-1 and PYY secretion to suppress appetite; CB2 activation modulates immune inflammation; PPARα activation drives fatty acid oxidation.	Emerging hypothesis based mainly on indirect mechanistic and receptor-based evidence. Current evidence is insufficient to define N-acylamides as validated therapeutic targets in pediatric obesity. They should be presented as exploratory candidates requiring pediatric clinical validation.	Li et al., 2024; Klag et al., 2026; Wei et al., 2023; Al-Daghri et al., 2021; Cheng et al., 2024; Cao et al., 2025; Mora-Godínez et al., 2025; Yin et al., 2024
5-Aminovaleric acid and other metabolites (5-AVA)	5-Aminovaleric acid	*Fusobacterium mortiferum, Parabacteroides goldsteinii*	Obesity is associated with elevated abundance of F. mortiferum and increased 5-AVA levels, which are tightly linked to the progression of obesity-related colorectal cancer. Pediatric obesity cohorts consistently exhibit widespread gut microbial dysbiosis and profoundly altered metabolite profiles.	Wnt/β-catenin pathway.	5-AVA activates the Wnt/β-catenin pathway through DKK2, thereby promoting colorectal tumorigenesis in the obese state, suggesting a detrimental metabolic bridge connecting obesity to malignant transformation. However, its direct causal role in adiposity accumulation *per se* remains to be elucidated. Additionally, 1,4-methylimidazoleacetic acid, associated with P. goldsteinii enrichment, has been observed in the context of obesity amelioration.	Mechanistic evidence (*in vivo*/vitro) validates the 5-AVA/Wnt-β-catenin pro-tumorigenic axis, with pediatric metabolomic studies providing associative support. Direct interventional pediatric data are lacking, positioning 5-AVA as a mechanistically supported obesity-cancer link rather than a clinically validated therapeutic target for pediatric obesity.	Zhang and Dang, 2022; de Cuevillas et al., 2022; Granato et al., 2025; Jardon et al., 2025; Nguyen Tran et al., 2025
Microbiota-modulating interventions related to metabolite output	Not a single metabolite; includes fiber-, chitosan-, metformin-, probiotic-, and lifestyle-related modulation of microbial metabolites	Depends on intervention-induced changes in SCFA-producing, tryptophan-metabolizing, bile-acid-transforming, and other functional bacteria	Interventions may partially restore dysbiotic microbiota and improve metabolite output profiles, but effects differ across individuals and developmental stages.	Indirect regulation of SCFA, aromatic amino acid-derived metabolite, bile acid, and endocannabinoid-like pathways.	These interventions support the clinical feasibility of targeting the microbiota-metabolite axis, but they do not prove that 4-HPAA, N-acylamides, or secondary bile acids themselves are validated pediatric therapeutic targets.	Randomized or non-randomized clinical intervention evidence; indirect support only. Pediatric and adolescent studies, including randomized, double-blind and pre-post dietary intervention designs, provide clinical support for microbiota modulation, but metabolite-specific causal evidence remains limited.	Mo et al., 2025; Visuthranukul et al., 2024b; Kilic Yildirim et al., 2023; Li et al., 2025; Du et al., 2025; Ayoub-Charette et al., 2023

**Table 4:** Summary of key functional gut microbiota and metabolic contributions related to obesity in children and adolescents.

Key functional microbial communities	Abundance changes under obesity	Main metabolic contribution	Core impact on host health	References
*Faecalibacterium prausnitzii*	Significantly reduce	Produces butyric acid and synthesizes propionic acid; produces N-acylamide regulatory factors; participates in BCAA transport regulation.	Anti-inflammatory, preserves the intestinal barrier, and fine-tunes immune homeostasis—potentially improving insulin sensitivity via BCAA modulation.	Yuan et al., 2021a; Newman et al., 2023; Moran-Ramos et al., 2021; Ahmed et al., 2021
*Bifidobacterium*	Reduce	Fermentable dietary fiber produces acetic acid and lactic acid; it participates in tryptophan metabolism to produce indole derivatives.	Maintains gut barrier, modulates immunity, suppresses pathogens, and ameliorates metabolic endotoxemia.	Agustina et al., 2025; Andriyas et al., 2025; Liang et al., 2024; Liang and Zhang, 2024
*Akkermansia muciniphila*	Reduce	Degrades mucin to produce propionic acid and acetic acid; regulates the thickness of the intestinal mucus layer.	Enhances barrier function, reduces endotoxin translocation, improves insulin sensitivity, and exerts anti-inflammatory effects.	Yuan et al., 2021b; Becken et al., 2021; Zhang Y. et al., 2025
*Flavonifactor plautii*	Speculative reduction	Metabolizes flavonol substances to produce 4-HPAA.	Through 4-HPAA, it activates ILC3s to strengthen the gut barrier; it downregulates intestinal fat absorption, curbing weight gain.	Jiang et al., 2025; Li Q. R. et al., 2025; Álvarez-Arraño and Martín-Peláez, 2021
*Roseburia*	Reduce	Mainly produces butyric acid	Supplies energy to colonic epithelium, sustains the intestinal barrier, and acts anti-inflammatory.	Akagbosu et al., 2025; Vázquez-Bolea et al., 2025
*Eubacterium*	Reduce	Produces butyric acid and participates in bile acid and cholesterol metabolism.	Preserves intestinal homeostasis, regulates host energy metabolism, and immune responses.	McCann et al., 2021
*Lactobacillus*	Some strains decrease	Produces lactic acid and acetic acid; some strains produce conjugated linoleic acid; regulates tryptophan metabolism.	Maintains an acidic gut environment, inhibits pathogens, and regulates immunity—may influence appetite via PYY secretion.	Liang et al., 2021; Agustina et al., 2025; Aragón-Vela et al., 2021; Mogos et al., 2025
*Bacteroidetes and Firmicutes*	Proportional imbalance	Mainly produces acetic acid and propionic acid; participates in carbohydrate and protein fermentation as well as bile acid metabolism.	Affects energy harvest; abundance closely tied to dietary fiber intake and SCFA production.	Gyarmati et al., 2021
*Clostridium cluster IV and XIVa*	Reduce	Core butyrate-producing bacterial groups, including *Faecalibacterium prausnitzii* and *Roseburia*.	Crucial for gut health and metabolic homeostasis; their depletion correlates with multiple metabolic disorders.	Carson et al., 2023; Akagbosu et al., 2022
*Lachnospiraceae*	Complex changes, some strains decrease	Contains various butyrate-producing bacteria, such as Roseburia; Participate *in carbohydrate fermentation*	Plays a key role in maintaining gut health and energy metabolism.	Akagbosu et al., 2025; Wang J. J. et al., 2024
*Ruminococcaceae*	Reduce	Produces acetic acid and butyric acid, involved in cellulose degradation.	Regulates intestinal immune tolerance and lipid absorption; SCFA-producing capacity is diminished in obese children.	Yuan et al., 2021a; Ma et al., 2023; Mörkl et al., 2025

## 4 Intervention strategies targeting microbial metabolites in childhood and adolescent obesity

**Figure 5:**
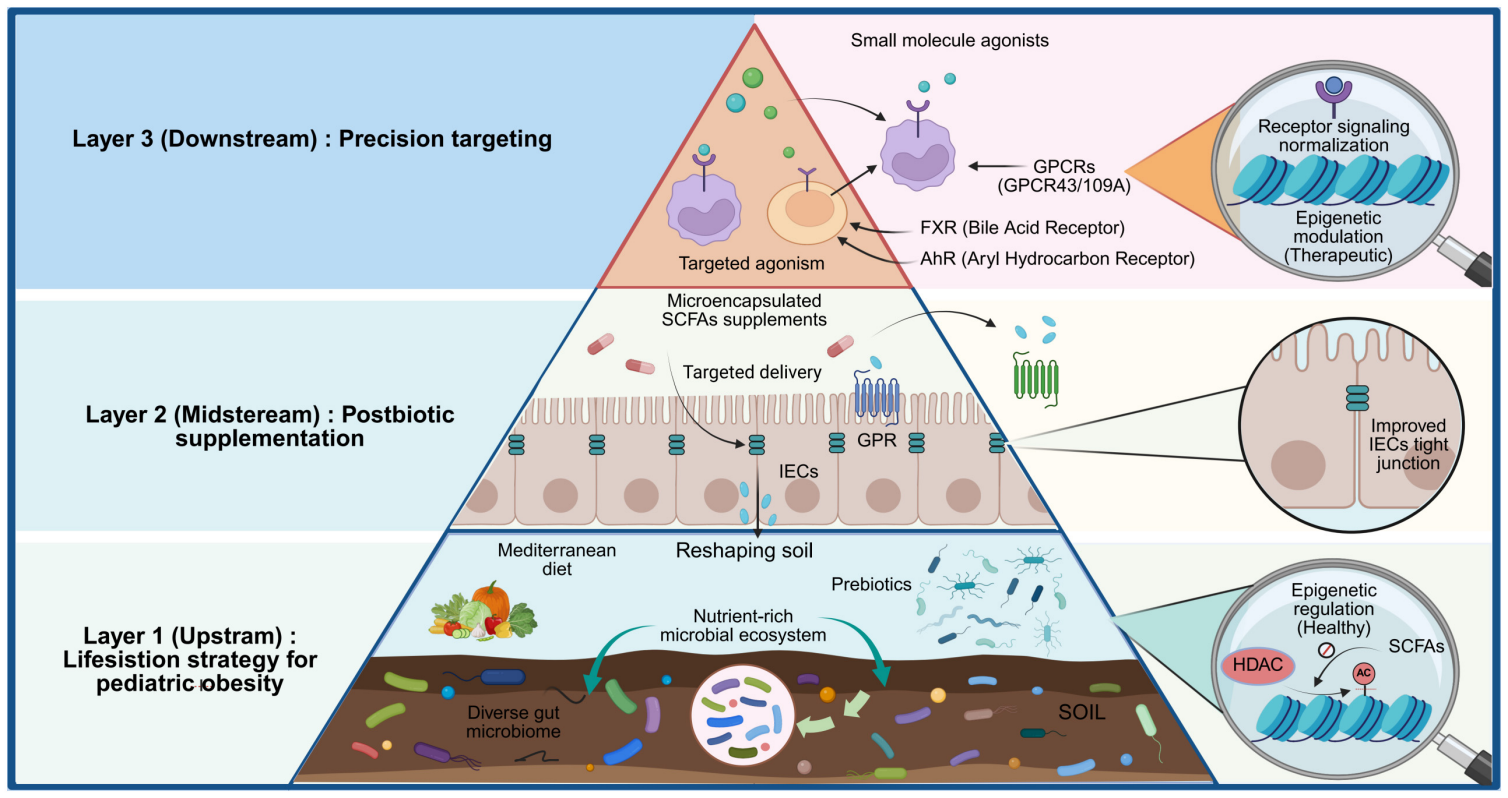
Multilevel intervention strategies targeting microbial metabolites in childhood and adolescent obesity. Created in BioRender. Qy, Z. (2026). https://BioRender.com/gioeshh.

Microbial metabolites—SCFAs, bile acids, AAA, and tryptophan metabolites, BCAA-related products, and trimethylamine N oxide (TMAO)—are involved in pediatric obesity-related metabolic regulation at different levels of evidence. SCFAs and selected bile acid-related signals may influence intestinal barrier function, inflammation, gut hormone secretion, lipid metabolism, and neuroendocrine pathways (Fiore et al., 2022; Koller et al., 2025; Nguyen Tran et al., 2025). Compared with simply restricting energy intake, microbiota–metabolite pathway-oriented strategies focus on the regulatory cascade of diet–microbiota–metabolites–host targets, which helps enhance personalized management and long-term adherence (Koller et al., 2025; Gawlik et al., 2021). This section categorizes relevant approaches into four major directions: upstream microbiota modulation, intermediate metabolite supplementation, downstream receptor intervention, and existing research limitations. The overall framework is presented in Figure 5.

### 4.1 Child-adapted prebiotic, high-fiber, and Mediterranean-style dietary strategies for modulating metabolite-producing microbiota

Prebiotics promote fermentation by beneficial bacteria, boosting SCFA output and thereby regulating fat storage, insulin sensitivity, and low-grade inflammation (Zhou et al., 2023; Pînzariu et al., 2025). They serve as selective substrates that enrich SCFA producers such as *Bifidobacterium* and *Faecalibacterium*, counteracting the dysbiosis and metabolic substrate depletion characteristic of high-fat, high-sugar, low-fiber diets. This approach raises gut propionate and butyrate levels and has shown potential in proof-of-concept studies to improve body composition and metabolic markers; for example, inulin, fructooligosaccharides, and (*in vitro*) stachyose all modulate the microbiota and metabolites of obese children, with inulin also influencing the gut–brain axis via microbial metabolite profiles (Visuthranukul et al., 2024b; Andriyas et al., 2025; Pi et al., 2024). The resulting metabolites not only promote PYY and GLP-1 secretion to regulate appetite, but may also influence body composition through putative gut–muscle axis pathways, although this remains largely hypothetical in humans, with particularly limited evidence in pediatric populations (Visuthranukul et al., 2024a; Yu X. et al., 2023; Panichsillaphakit et al., 2025). Fiber tolerance varies; therefore, gradual dose escalation and selection of appropriate fiber types are essential to minimize bloating and pain, and attention to potential biphasic effects is warranted (Basuray et al., 2024).

Dietary pattern modification is the main way to reshape the intestinal metabolic microenvironment. Increased intake of whole grains, legumes, fruits, vegetables, and nuts provides many fermentable substrates, increases the production of SCFAs and beneficial metabolites, and limits harmful metabolic pathways. In contrast, limiting ultra-processed foods and sugar-sweetened beverages can lower metabolic risks and improve microbial metabolic profiles (Calcaterra et al., 2025, 2024; Lane et al., 2024). Practical measures, such as choosing high-fiber staple foods, selecting healthy snacks, and using family-based shopping guidance that matches children's tastes, can make the intervention easier to carry out and support long-term adherence (Aqeel et al., 2025). The Mediterranean diet, which is rich in fiber and polyphenols, supports a stable, anti-inflammatory, and metabolically resilient microbiota. It shows potential for pediatric obesity and related comorbidities and can be maintained in both home and school settings (Bagheri et al., 2022; Deligeorgopoulou et al., 2025). Such holistic dietary interventions work as long-term regulation of the gut ecosystem. They may increase microbial diversity and SCFA-related metabolic capacity through more diverse dietary fiber intake (Yu et al., 2025; Basuray et al., 2024; Yang et al., 2024). Importantly, the preventive effect of dietary fiber on childhood obesity is microbiota-dependent, underscoring the central role of the high-fiber diet–microbiota–metabolite axis (Yu et al., 2025).

Modern gut microbiota research has evolved from taxonomic profiling to function-oriented analyses focused on metabolites. Quantitative analyses reveal substantial inter-individual variation in the efficiency with which a given substrate is converted into metabolites, calling for upstream strategies that match substrate, microbial function, and metabolite output (Arnoldini et al., 2025; Gawlik et al., 2021). Prebiotic supplementation and high-fiber/Mediterranean diets are thus complementary: prebiotics are being investigated for early clinical correction of specific metabolic deficits, while high-fiber dietary patterns form the foundation for long-term health management (Cho, 2021; Zhou et al., 2024). Combined use of these approaches has the potential to ameliorate SCFA insufficiency induced by dysbiosis, and may help restore the protective effects of SCFAs in preserving mucosal integrity, regulating energy metabolism and restraining chronic low-grade inflammation (Zhang and Dang, 2022; Li S. et al., 2025). Gut microbial disturbance is generally more severe in obese children than in adults, offering a broader time frame for microbiota modulation. Meanwhile, all interventions need to fully consider age-related physiological features (Yu Z. J. et al., 2023; Carson et al., 2023). Collectively, although prebiotic interventions are conceptually attractive and supported by mechanistic plausibility, they remain predominantly at the stage of experimental investigation; large-scale pediatric efficacy and safety trials are needed before clinical translation (Wu et al., 2025; Fahim et al., 2025). To put these strategies into practice, precision metabolite-based interventions should be combined with microbiota ecosystem cultivation. This can be done through new food design and systematic nutrition education that closely fit children's dietary preferences.

### 4.2 Postbiotic supplements in children and adolescents: dose optimization for SCFAs and safety of targeted metabolite formulations

Dysfunction of the gut microbiota–immune–metabolic axis is an important component of childhood obesity pathophysiology, rather than a single main driver. Given the special metabolic flexibility in pediatric populations, postbiotic interventions need to fit developmental characteristics. They should not simply copy treatment plans used for adults. Children with obesity have been reported in some studies to show altered abundances of SCFA-producing bacteria, which may affect SCFA-related metabolic and immune signaling. For this reason, supplementary SCFA blends are designed to adjust SCFA availability and support GPR41/43-related regulation of adipogenesis, inflammation, and gut hormone signaling, rather than as interventions that directly rebuild host signaling. These blends are hypothesized to mainly affect energy-sensing pathways within the gut–brain axis. For instance, inulin can regulate microbial metabolites and has been associated with changes in neural activity, neurotransmitter metabolism, and eating behavior, although these associations are preliminary and derived from a limited number of observational studies (Li S. et al., 2025; Andriyas et al., 2025). A stratified dosing strategy has been proposed. This strategy divides patients into three levels based on BMI and insulin resistance: baseline regulation, metabolic correction, and homeostasis maintenance. This approach allows personalized dose adjustment through real-time monitoring (Luzzi et al., 2024). Safety considerations are also important. Encapsulation technologies can reduce mucosal irritation related to SCFAs, and long-term effects on growth hormone secretion have been reported (Granato et al., 2025). Pediatric products also need to solve problems such as poor taste and low delivery efficiency. They should start from low doses and then increase gradually (Basuray et al., 2024). The proportion of acetate, propionate, and butyrate should be adjusted according to treatment goals, such as improving insulin sensitivity. Additionally, the combined efficacy of SCFA supplements with dietary modification and physical activity still needs further confirmation (Cho, 2021; Lee et al., 2023; Islam et al., 2023).

Apart from SCFAs, supplementation with specific metabolite-based postbiotics may offer a theoretical advantage over live probiotics by circumventing colonization instability and directly providing defined functional compounds. Microbe-derived IPA has anti-obesity effects by regulating intestinal immunity (Jiang et al., 2025). 4-HPAA, whose levels are lower in obese children, shows promising effects in limiting body weight gain (Li Q. R. et al., 2025). Research on Fusobacterium mortiferum and its metabolite 5-aminovaleric acid has improved our understanding of how microbial metabolites influence metabolic health (Deng et al., 2025). Tryptophan-related metabolites are associated with cardiometabolic risk indicators in adolescents. This supports their value in risk stratification and targeted intervention (Rivera et al., 2025). Conversely, TMAO and its precursors are closely related to childhood obesity. This suggests that interventions should not only add beneficial metabolites but also reduce the production of harmful ones (Li et al., 2025). Multi-omics analyses also support these strategies by showing links between pediatric gut microbiota and different lipidomic profiles or newly identified microbial metabolites. This helps identify possible therapeutic targets (Wei et al., 2023; Mora-Godínez et al., 2025; Du et al., 2025). Although postbiotic approaches are mechanistically attractive, they remain predominantly at the preclinical or early-phase clinical stage; given that children's metabolic systems are still developing, rigorous dose-finding studies and long-term safety evaluations in pediatric populations are urgently needed before clinical translation (Wu et al., 2025; Fahim et al., 2025). These longitudinal studies should clarify their effects on physical growth and development, avoid disruption of endogenous metabolic pathways, and confirm whether this type of supplementation is reasonable for young populations (Solito et al., 2021).

### 4.3 Metabolite-receptor-targeted agents tailored to pediatric physiology

Downstream therapeutic strategies focus more on precise control of host receptor activity rather than directly supplementing microbial metabolites. These agents are designed to mimic or enhance endogenous metabolite signaling; in principle, they may circumvent certain dependencies on the gut microbiota, though this theoretical advantage remains to be substantiated in pediatric populations (Zhou et al., 2023; Pînzariu et al., 2025). In pediatric populations, drug selectivity and dosage schemes need to match age-specific pharmacokinetic features and receptor expression patterns. Gut-localized GPCR agonists with limited systemic absorption are preferred because they can provide anti-inflammatory and metabolic benefits. At the same time, their long-term effects on bile acid circulation and the neuroendocrine axis need systematic evaluation (Li S. et al., 2025; Giannini et al., 2022). A number of therapeutic targets have been confirmed. Experimental studies suggest that aromatic amino acid and tryptophan-derived metabolites may influence immune and epithelial barrier pathways through receptors such as AHR, but this does not establish AHR agonists as validated anti-obesity drugs in children (Jiang et al., 2025). Targeting FXR and TGR5 may participate in glucose, lipid, inflammatory, and gut hormone regulation; however, the effects of these pathways are tissue-specific and context-dependent, and pediatric therapeutic use remains investigational (Giannini et al., 2022; Mancera-Hurtado et al., 2023). Acid-sensing ion channels have been shown to be associated with obesity, making metabolic-sensing receptors possible new targets for drug discovery, but these associations do not yet confirm them as clinically actionable targets in children (Shearer et al., 2025). On this basis, bile acid receptor modulators with pediatric-adapted dosages are being explored as experimental candidates. AHR agonists and agents targeting 4-HPAA-associated receptors are also being studied (Giannini et al., 2022; Li Q. R. et al., 2025; Mancera-Hurtado et al., 2023). For eventual clinical translation, it will be necessary to build age-specific receptor pharmacology databases and combine them with blood metabolite profiling to support personalized therapy. Formulations such as chewable tablets and oral solutions can improve treatment adherence. Large-scale, long-term pediatric clinical trials are still needed to determine whether these receptor-targeted agents are safe, effective, and mechanistically appropriate for children and adolescents with obesity (Jardon et al., 2025; Solito et al., 2021; Verma et al., 2021).

### 4.4 Multi-omics technologies for identifying and validating microbial metabolite pathways in pediatric obesity

The focus of pediatric obesity research was shifted from 16S rRNA sequencing-based single-microbiota profile analysis (Jaimes et al., 2021; Chen et al., 2021; Zhou R. J. et al., 2025) to an integrated multi-omics paradigm. Because the mechanisms of disease were not elucidated by microbial metabolite analysis alone, multi-level omics data were integrated for the analysis of host-microbiota-immune interactions. 16S rRNA sequencing was combined with ^1^H NMR metabolomics for large-scale sample screening (Jaimes et al., 2021). Shotgun metagenomics was utilized for the identification of microbial species and functional genes, and obesity-related pathway genes—including those associated with carbohydrate, amino acid, and dietary fiber fermentation, as well as endotoxin synthesis—were investigated (Zhou et al., 2022; Pastor-Villaescusa et al., 2021). Metatranscriptomics and metaproteomics were applied for the detection of microbial metabolic activity and expression levels of inflammation-related genes and proteins (Calabrese et al., 2021; Liu et al., 2025). Metabolomics and lipidomics were based on chromatography-mass spectrometry and nuclear magnetic resonance platforms; metabolites—including short-chain fatty acids, bile acids, trimethylamine N-oxide, and acylcarnitines—were identified and quantified, and were associated with inflammation, insulin resistance, metabolic dysfunction-associated steatotic liver disease, and cardiometabolic impairment (Yin et al., 2024; González-Domínguez et al., 2024; Hou et al., 2023). Multi-omics was utilized for the integration of metagenomics, metabolomics, lipidomics, proteomics, immune indices, and clinical phenotypes, and microbial-metabolite-protein-immune interaction networks were constructed. Causality and correlation of microbial markers were distinguished through machine learning, mediation effects, and Mendelian randomization, and microbial gene-effector molecule-immune metabolic regulatory networks were established for the subtyping of obesity (Yin et al., 2024; Liu et al., 2025; Lin et al., 2025); the ‘microbiota remodeling-metabolic disturbance-immune imbalance' cascade was evidenced (Deligeorgopoulou et al., 2025; Chan et al., 2026; Luo et al., 2025). Heterogeneity in populations, technologies, and analyses was identified, and result reproducibility was limited. Limitations—including a prevalence of cross-sectional designs, insufficient sample sizes, and a lag in the development of single-cell and spatial omics—were noted in existing studies (Becken et al., 2021; Marabita et al., 2022; Scanlon et al., 2025). Large-scale longitudinal cohorts and deep learning multi-omics algorithms were utilized for subsequent research, and the molecular mechanisms of microbiota-immune crosstalk were examined to provide evidence for targeted interventions, such as fecal microbiota transplantation (Morgado et al., 2023; Gawlik et al., 2021; Wilson et al., 2025).

### 4.5 Tissue-specific metabolite dynamics, interindividual variability and complex regulatory networks

Despite their promise, metabolite-targeted interventions still face three main bottlenecks. First, accurate detection of metabolite levels across different tissues is still a major technical barrier. Most current studies use fecal metabolomics analysis, but this method has limited capacity to accurately reflect metabolite concentrations in the portal vein or target tissues. Nevertheless, fecal metabolomics still provides valuable insights into gut microbial activity and metabolic state. Notably, compartmental differences in metabolites are closely related to the development of local lesions, including tissue-specific insulin resistance (Jardon et al., 2025; Luo et al., 2025). Pediatric multi-omics work on MASLD also shows the complex link between circulating gut-derived metabolites and liver pathology (Du et al., 2025; Lin Y. C. et al., 2025). Compared with adults, children and adolescents have less mature metabolic systems. This leads to different patterns in drug absorption, distribution, metabolism, and excretion. These differences make monitoring more difficult and show the urgent need for minimally invasive detection techniques that can measure tissue-specific metabolites (Li et al., 2024; Xia et al., 2025).

Secondly, gut microbiota and its metabolic products show clear differences between individuals and also change over time. This microbial ecosystem develops together with the host, and its composition is affected by pubertal hormones, ethnic background, and early feeding patterns (Huang and Roth, 2021; Carson et al., 2023; Belkova et al., 2025). Distinct age-related and population-specific characteristics make outcome prediction more difficult. Typical examples include abnormal methane metabolism seen in obese children from certain regions and unique microbial changes found in Chinese populations (Gawlik et al., 2021; Wang J. J. et al., 2024; Zhou et al., 2022). Such differences mean that intervention plans need to be made based on baseline microbiota profiling.

Thirdly, explaining complex regulatory networks while avoiding off-target effects is a major challenge. Microbe-host interactions involve many factors, including microbiota, host genome, immunity, and environmental factors. Nevertheless, most existing research still focuses on single metabolites or single signaling pathways, and the full regulatory network has not been fully described. Moreover, while multi-omics approaches are powerful for uncovering correlational patterns and identifying candidate biomarkers, they cannot independently establish causative mechanisms; functional validation in appropriate models remains essential (Vander Wyst et al., 2021; Du et al., 2025; Lin Y. C. et al., 2025). Interventions may also cause persistent unintended adverse effects. To tackle this issue, researchers need to combine multi-omics data to identify key regulatory nodes and carry out long-term observational studies to clarify how early-life gut microbiota shape long-term health outcomes. Such efforts will help turn correlational findings into validated therapeutic targets (Table 5; Yin et al., 2024; Dinleyici, 2025).

**Table 5:** Microbial metabolite-targeted intervention strategies for childhood and adolescent obesity.

Intervention level	Core strategy	Specific intervention methods	Expected biological effect	Applicable people	Transformation challenge	References
Upstream intervention	Change the microbiota and improve the metabolite output profile	Supplement prebiotics, such as inulin and fructo-oligosaccharides; follow a high-fiber diet and a Mediterranean diet; and reduce ultra-processed food intake.	Selectively increases gut bacteria that produce beneficial metabolites, such as SCFAs and 4-HPAA; raises gut SCFA levels; and improves the overall metabolome.	First-line basic approach for children and adolescents at risk of obesity.	Fiber tolerance varies greatly between individuals; children's dietary preferences need to be considered; responses may differ between children.	Bagheri et al., 2022; Orbe-Orihuela et al., 2022; Deligeorgopoulou et al., 2025; Mo et al., 2025; Visuthranukul et al., 2024b; Andriyas et al., 2025; Basuray et al., 2024; Yang et al., 2024
Midstream intervention	Directly supplement key metabolites	Develop SCFA compound preparations designed for children; supplement safe preparations, such as 4-HPAA and indole-3-propionic acid; explore ways to reduce the production of harmful metabolites.	Bypasses differences in the microbiota and delivers beneficial signals directly; quickly regulates gut immunity and barrier function; allows more precise intervention.	Children with severe dysbiosis; children who do not respond to upstream interventions; and children who need rapid metabolic correction.	Finding safe and effective doses for children; solving problems with stability, taste, and delivery; avoiding interference with the body's own pathways.	Rivera et al., 2025; Granato et al., 2025; Pînzariu et al., 2025; Li Q. R. et al., 2025; Li et al., 2025; Luzzi et al., 2024; Solito et al., 2021
Downstream intervention	Precise regulation of host receptor function	Develop receptor-targeted formulations for children, such as FXR/TGR5 agonists and CB1 antagonists.	Does not depend on microbiota structure or metabolite levels; directly stabilizes, mimics, or blocks useful signals; reduces the impact of differences in microbiota between individuals.	Individuals with disturbances in specific metabolic pathways, such as abnormal bile acid metabolism or an imbalance in the endogenous cannabinoid system.	for age-stratified pediatric receptor pharmacology database; assessing long-term effects on the developing neuroendocrine axis.	Zhou et al., 2023; Jiang et al., 2025; Li S. et al., 2025; Chan et al., 2026; Giannini et al., 2022; Al-Daghri et al., 2021; Aragón-Vela et al., 2021; Mancera-Hurtado et al., 2023; Shearer et al., 2025

### 4.6 Current technical limitations and research bottlenecks in gut microbiota and metabolite studies

Although research on the gut microbiome and metabolites was expanded rapidly, several methodological challenges were identified as core bottlenecks that limited the interpretation and reproducibility of study results. First, dietary composition was confirmed as a primary confounding factor, as total energy intake, fiber content, ultra-processed food consumption, and dietary patterns were verified to reshape microbial composition and metabolite levels (Verduci et al., 2021a; Bagheri et al., 2022; Zheng et al., 2025). Furthermore, variables—including physical activity, sleep rhythms, antibiotic and other medication use, pubertal development, and early-life environmental factors —were observed to exert pressure on the microbiota-metabolite profile independently of disease status (Barata et al., 2025; Celik and Yesildemir, 2025; Chu et al., 2025). Second, heterogeneity across studies was attributed to differences in demographic characteristics, such as age, geographic region, obesity criteria, and comorbidities, as well as experimental protocols, including sampling, preservation, sequencing platforms, and bioinformatics pipelines; batch effects were induced by the lack of standardized operations, and contradictory conclusions were generated (Gawlik et al., 2021; Liu et al., 2025; He et al., 2025). Third, causal inference was constrained by study design; observational or cross-sectional designs were employed, and the establishment of temporal causality was hindered by the scarcity of long-term longitudinal cohorts (He et al., 2025; Pan et al., 2026). In mechanistic validation, differences in immune-metabolic programming between species and off-target effects following microbiota transplantation were noted in animal models (Chen et al., 2022; DeLeon et al., 2025; Jeong et al., 2024). In clinical translation, defects—including insufficient sample sizes in randomized controlled trials, the absence of baseline microbiome stratification, and the lack of long-term safety assessments for fecal microbiota transplantation in adolescents—were recorded (Pastor-Villaescusa et al., 2021; Fatahi et al., 2025; Wang et al., 2025). Standardized sampling, integrated multi-omics research, large-scale longitudinal birth cohorts, and cross-regional collaborations were identified as pathways for future research, and causal inference was strengthened to facilitate the transition from correlation-based descriptions to the identification of evidence chains with clinical translational value (Lin et al., 2025; Shi et al., 2025; Suárez-Cortés et al., 2025).

## 5 Summary and future perspectives

**Figure 6:**
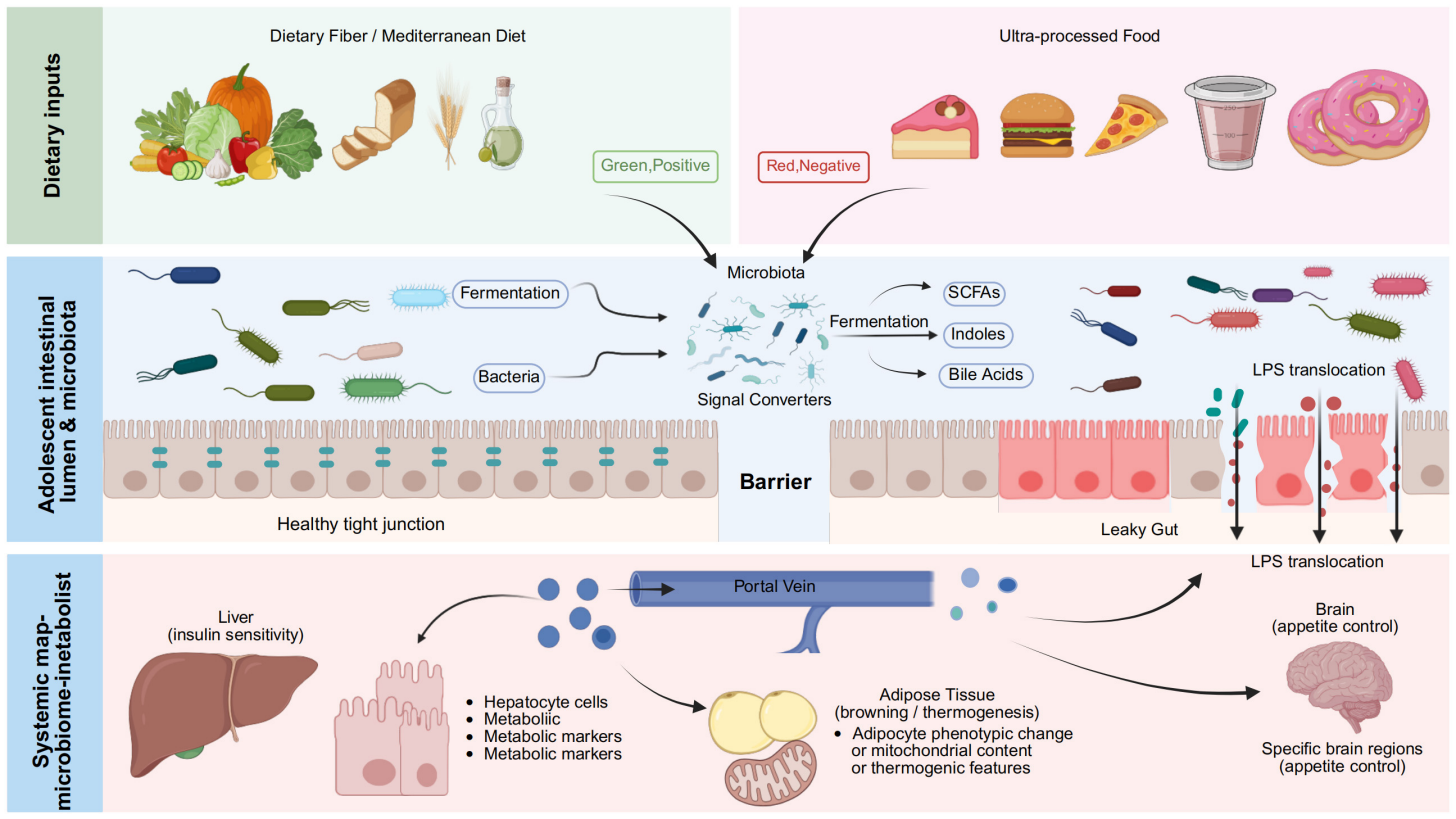
Five-node cascade regulatory pathway from diet to metabolic organs in children and adolescents. Created in BioRender. Qy, Z. (2026). https://BioRender.com/fjyobzy.

Childhood and adolescent obesity develops from the combined effects of gut dysbiosis, metabolic reprogramming, and chronic low-grade inflammation during key developmental periods. It does not result only from simple energy imbalance (Cho, 2023; Mindru et al., 2025). Gut microbial metabolites should be viewed as a heterogeneous group of candidate mediators rather than as equivalent components with identical biological roles or evidence strength. SCFAs have relatively strong mechanistic support for effects on epithelial barrier integrity, gut hormone secretion, immune regulation, and energy metabolism (Fiore et al., 2022; Li S. et al., 2025). In contrast, indole derivatives and secondary bile acids represent emerging immune-metabolic signaling candidates whose roles are mediated mainly through AHR- and FXR/TGR5-related pathways, but their direct pediatric therapeutic validation remains limited (Giannini et al., 2022; Rivera et al., 2025). 4-HPAA is a promising obesity-associated microbial metabolite, but current evidence is still based mainly on observational associations and experimental validation rather than robust pediatric intervention data (Li Q. R. et al., 2025). Therefore, these metabolites should not be interpreted as having the same level of evidence or the same therapeutic maturity. Collectively, these metabolites affect metabolic and immune balance through a multi-node network that connects dietary substrates, microbiota, metabolites, immune cells, and metabolic organs (Zhou et al., 2023; Jiang et al., 2025; Zhang and Dang, 2022). Figure 6 shows our integrated “dietary substrate–gut microbiota–metabolites–immune cells–metabolic organs” framework. This framework highlights multi-level signaling pathways and feedback loops from environmental input to host response and provides a systematic view of disease development and intervention targets.

### 5.1 Evidence-graded and developmentally informed roles of microbial metabolites in the axis

A unified logic supports this axis: specific metabolites turn microbiota structure into host immune regulation, and they can either promote or limit obesity (Zhou et al., 2023). This shared framework operates through complementary mechanisms—ranging from barrier preservation and immune cell polarization to systemic anti-inflammatory signaling—that collectively maintain host metabolic homeostasis (Jiang et al., 2025; Rivera et al., 2025; Czarnowski et al., 2023).

Metabolites also form a connected and complementary network. In energy metabolism, SCFAs regulate appetite and thermogenesis, whereas 4-HPAA reduces fat deposition by lowering the expression of intestinal fatty acid transporters (Visuthranukul et al., 2024a; Li Q. R. et al., 2025). From a developmental perspective, infants depend on acetate and propionate for rapid growth, while puberty is marked by higher levels of butyrate and secondary bile acids, which help maintain mature metabolic homeostasis (Carson et al., 2023; Oksanen et al., 2025). In terms of intervention strategies, prebiotics can increase SCFA levels and help restore intestinal barrier function. Combined supplementation with indole-3-propionic acid can further improve insulin sensitivity and reduce inflammation, which may create synergistic effects (Mo et al., 2025; Luzzi et al., 2024). SCFAs act as core mediators that connect immunity and metabolism. AAA metabolites, such as 4-HPAA and indole derivatives, work as intermediate signaling molecules, and abnormal BCAA metabolism is strongly linked to insulin resistance in adolescents (Del Chierico et al., 2021; Rivera et al., 2025; Li Q. R. et al., 2025). Bile acids mainly regulate the liver and adipose tissue. They complement SCFA-mediated gut mucosal protection and extend the regulatory network across several organs (Giannini et al., 2022; Zöggeler et al., 2025; Mancera-Hurtado et al., 2023).

This functional specialization provides the necessary bridge from molecular mechanisms to intervention strategy. A metabolite-related mechanism becomes clinically meaningful only when it satisfies three conditions. First, the disturbance should be measurable and reproducible in pediatric obesity, such as reduced SCFA-producing capacity, abnormal amino-acid-derived metabolites, or altered bile-acid profiles (Li S. et al., 2025; Yu X. et al., 2023; Wei et al., 2023). Second, the disturbance should be linked to an actionable phenotype, including impaired barrier function, cytokine activation, insulin resistance, hepatic steatosis, abnormal appetite regulation, or adipose dysfunction (Newman et al., 2023; Fiore et al., 2022; Zöggeler et al., 2025). Third, the proposed intervention should match the developmental stage and safety requirements of children and adolescents, because the microbiota, immune system, endocrine axis, and eating behavior are still changing during growth (Jian et al., 2021; Gawlik et al., 2021; Fahim et al., 2025). This stepwise logic prevents an abrupt transition from receptor-level mechanisms to clinical treatment and instead supports a biomarker-guided, developmentally appropriate intervention framework.

A defining feature of pediatric obesity is its clear developmental stage specificity, which creates an important window for early intervention. Abnormal gut microbial colonization in early life can lead to long-term metabolic risks, and hormonal changes during puberty interact with microbial metabolites to affect the progression of obesity (Huang and Roth, 2021; Carson et al., 2023). Meanwhile, the gut microbiota in developing individuals has high plasticity, which allows metabolite profiles to be quickly reshaped through dietary adjustment and prebiotic intervention (Gawlik et al., 2021). However, this plasticity should be translated into clinical practice in a graded rather than immediate manner. Upstream strategies, including high-fiber dietary patterns, Mediterranean-style diets, and prebiotic or probiotic approaches, should be prioritized because they reshape microbial ecology and increase beneficial metabolite production with relatively low risk (Deligeorgopoulou et al., 2025; Mo et al., 2025; Visuthranukul et al., 2024b). Midstream strategies, such as pediatric-oriented SCFA-supporting formulations, postbiotics, or candidate metabolite supplementation, may be considered when specific metabolite insufficiency or severe dysbiosis is identified, but pediatric dose, delivery, tolerance, and long-term safety still require validation (Pînzariu et al., 2025; Luzzi et al., 2024; Solito et al., 2021). Downstream receptor-targeted approaches, including FXR/TGR5- or endocannabinoid-related modulation, should be framed as future precision options for selected subgroups rather than immediate general recommendations, because their long-term effects on the developing neuroendocrine and immune systems remain insufficiently defined (Fahim et al., 2025; Mancera-Hurtado et al., 2023; Shearer et al., 2025). Therefore, the clinical value of microbial metabolites lies not in proposing one universal treatment, but in using metabolite profiles to connect mechanism, phenotype, developmental stage, and intervention intensity. This framework offers a clearer rationale for precision prevention and treatment of childhood and adolescent obesity (Yu Z. J. et al., 2023; Carson et al., 2023; Gawlik et al., 2021).

### 5.2 The “diet–microbiota–metabolites–immune cells–metabolic organs” cascade

We propose a five-node framework with SCFAs and newly identified metabolites acting as core signaling mediators. This model redefines pediatric obesity as a systemic disorder characterized by developmentally vulnerable immune-metabolic crosstalk, instead of a mere imbalance between energy intake and expenditure (Baranowski and Motil, 2021; Verduci et al., 2021a). The framework includes five connected layers: diet, gut microbiota, microbial metabolites, immune cells, and metabolic organs. These layers should not be viewed as isolated components. Instead, each layer converts signals from the previous layer into biological changes in the next layer, thereby forming a continuous cascade that links dietary exposure to metabolic dysfunction (Carson et al., 2023; Jian et al., 2021; Yang et al., 2024).

At the top of the cascade, diet functions as the upstream driving factor. High-fiber substrates including inulin, fructooligosaccharides, fruit fibers, vegetable fibers and whole-grain fibers selectively nourish beneficial bacteria capable of producing SCFAs and 4-HPAA, such as Akkermansia muciniphila and Faecalibacterium prausnitzii, and remodel metabolite profiles toward a healthier phenotype (Visuthranukul et al., 2024b; Andriyas et al., 2025; Li Q. R. et al., 2025). In contrast, diets high in sugar and fat, along with sugar-sweetened beverages and ultra-processed foods promote pathobiont proliferation, enhance harmful metabolite production, and suppress beneficial microbial fermentation (Prodam et al., 2025; Lane et al., 2024; Navajas-Porras et al., 2022). Notably, certain dietary fibers exert attenuated biological effects in obese children compared with animal experiments, which further demonstrates the unique developmental characteristics of pediatric population (Basuray et al., 2024; Czarnowski et al., 2024). Mediterranean-style patterns support less pro-inflammatory metabolite output (Deligeorgopoulou et al., 2025).

The gut microbiota acts as the central hub of this regulatory cascade. Children have lower gut microbial diversity than adults, the degree of dysbiosis is more pronounced in obese pediatric populations (Yu Z. J. et al., 2023). Early microbial colonization, which is shaped by breastfeeding patterns and delivery modes, affects long-term susceptibility to obesity. Lifestyle factors during puberty also further change gut microbial composition and function (Dinleyici, 2025; Zhou C. H. et al., 2025; Santarossa et al., 2021). Therefore, the microbiota represents the first biological interface between external dietary exposure and internal immune-metabolic regulation. In obese children, impaired fiber fermentation and reduced microbial antioxidant capacity can disturb metabolite production and lead to an abnormal metabolite profile (Arnoldini et al., 2025; Cheng et al., 2024). Metabolites transmit signals across the intestinal barrier and via the circulation to immune cells and metabolic organs. Beneficial metabolites activate anti-obesity and anti-inflammatory pathways; when the barrier is compromised, pathogenic metabolites (e.g., endotoxins) translocate, triggering systemic inflammation, telomere shortening, and metabolic dysfunction (Al-Daghri et al., 2021; Pînzariu et al., 2025; Ayala-García et al., 2024). In this way, metabolites serve as the molecular bridge between gut microbial dysbiosis and host immune-metabolic responses. They also participate in communication along the gut–brain, gut–liver, gut–muscle, and gut–gonad axes, thereby extending the cascade beyond the intestine (Andriyas et al., 2025; Visuthranukul et al., 2022).

Immune cells are key nodes in this network. Beneficial metabolites promote Tregs, suppress pro-inflammatory macrophages and mucosal-associated invariant T cells, and protect insulin signaling (Newman et al., 2023; Li et al., 2022). Pathogenic metabolites drive chronic inflammation and insulin resistance (Yuan et al., 2023; Del Chierico et al., 2021; Ayala-García et al., 2024). The developing immature immune system is highly responsive to microbial metabolites, making early-life dysregulation a strong contributor to progression from obesity to metabolic syndrome (Carson et al., 2023; Koller et al., 2025; Zhou et al., 2022). The signals ultimately reach downstream effector organs-the liver, adipose tissue, pancreas, and hypothalamus are downstream effectors. Metabolite–immune disturbances cause hepatic steatosis, adipose inflammation and hypertrophic adipocytes, β-cell dysfunction, and disrupted appetite control, synergistically sustaining the obese phenotype (Visuthranukul et al., 2022; Cai et al., 2025; Ismail et al., 2025). These organ-level effects reinforce one another and help sustain the obese phenotype. Therefore, the five-node cascade provides a stepwise explanation of how dietary patterns can be converted into microbial dysbiosis, metabolite imbalance, immune activation, and finally multi-organ metabolic dysfunction in childhood and adolescent obesity.

### 5.3 Integrating multi-omics to uncover developmental mechanisms, validate causality, and develop personalized metabolite-based interventions

Integrating metabolomics with single-cell transcriptomics, proteomics, and epigenomics will help pinpoint the metabolite profiles and target cell subsets characteristic of different developmental stages, including the interplay between pubertal hormones and microbial metabolites and the specific features of metabolite transport across an immature intestinal barrier (Yin et al., 2024; Dinleyici, 2025; Zhou C. H. et al., 2025). Large-scale longitudinal cohorts tracking diet, microbiota, metabolites, and obesity from early childhood through adolescence are needed to identify critical windows and development-specific regulatory nodes (Jian et al., 2021; Zheng et al., 2025; Cai et al., 2025).

Establishing stronger causal inference requires moving beyond traditional correlational and cross-sectional study designs. Mendelian randomization can support causal inference between microbial metabolites and pediatric obesity while reducing the influence of confounding factors (Li et al., 2024; Lu et al., 2024). In animal models, fecal microbiota transplantation (FMT) combined with metabolite supplementation, or microbial metabolite knockout, can help validate the causal roles of specific metabolites. However, FMT in children remains experimental, and its application for causal validation is not yet established (Wilson et al., 2025; La Rosa et al., 2025). Targeted gene editing of microbial metabolic pathways or host metabolite receptors can further improve the accuracy of causal testing (Jiang et al., 2025; Cheng et al., 2024). Ultimately, large-scale multicenter RCTs are indispensable to evaluate the clinical efficacy and safety of metabolite-targeted strategies in pediatric populations (Wu et al., 2025; Fahim et al., 2025). Nevertheless, RCTs mainly demonstrate whether an intervention produces a treatment effect under controlled conditions. They do not, by themselves, definitively prove the underlying biological mechanism. To support mechanistic interpretation, future RCTs should include pre-specified mechanistic endpoints, such as microbial functional profiling, fecal and circulating metabolomics, inflammatory markers, insulin sensitivity, and immune-metabolic readouts. Notably, inconsistencies between preclinical animal data and pediatric clinical outcomes show the urgent need to develop age-matched experimental models that fit childhood developmental characteristics (Czarnowski et al., 2024).

For the advancement of personalized intervention strategies, machine learning-based microbiota-metabolite classification systems should be built for pediatric obesity. These systems can help achieve precise patient stratification and identify therapeutic targets for specific subtypes (Squillario et al., 2023; Belkova et al., 2025; Wang J. J. et al., 2024; Wang M. P. et al., 2024). It is critical to develop pediatric-adapted metabolite-oriented interventions, including improved SCFA compound formulations, standardized 4-HPAA supplementation, and microencapsulation techniques for targeted intestinal delivery. Innovative formulation technologies are also needed to improve taste acceptability and *in vivo* bioavailability (Li Q. R. et al., 2025; Verma et al., 2021). Individualized diet–exercise regimens can reshape metabolite-producing microbial communities at the basic level. When combined with long-term cohort trials to evaluate clinical safety, efficacy, and mechanism-related biomarkers, they become an essential part of precise management (Morgado et al., 2023; Wilson et al., 2025; Morán-Ramos et al., 2022). Given the high complexity of microbial immune-metabolic networks, multi-target combinatorial interventions deserve further study. These synergistic strategies include prebiotic–probiotic–postbiotic three-part combinations to jointly regulate microbial composition and metabolic function (Álvarez-Arraño and Martín-Peláez, 2021; Luzzi et al., 2024; Zhang L. et al., 2025); metabolite-targeted agents combined with diet, exercise, and pharmacotherapy into multidimensional programs (Glaros et al., 2025; Aqeel et al., 2025; Sun W. et al., 2025); strategies that address both obesity and common comorbidities (Kopańska et al., 2025; Lei et al., 2025; Wang L. et al., 2024); and digital tools that support real-time monitoring and dynamic adjustment to improve precision and adherence (Marabita et al., 2022).

For clarity, Table 6 lists the main abbreviations used in the gut microbiota–immune–metabolic axis, intervention targets, and related molecular mechanisms.

**Table 6:** List of abbreviations.

Abbreviation	Full name
SCFAs	Short-chain fatty acids
GPCRs	G protein-coupled receptors
HDAC	Histone deacetylase
AAA	Aromatic amino acid
FXR	Farnesoid X receptor
TGR5	Takeda G protein-coupled receptor 5
TNF-α	Tumor necrosis factor-α
IL-6	Interleukin-6
GPR41	G protein-coupled receptor 41
GPR43	G protein-coupled receptor 41
PYY	Peptide YY
GLP-1	Glucagon-like peptide-1
HPA	Hypothalamic–pituitary–adrenal
AHR	Aryl hydrocarbon receptor
Wnt	Wingless-related integration site
JAK/STAT	Janus kinase/signal transducer and activator of transcription
BCAA	Branched-chain amino acids
Treg	Regulatory T cells
NF-κB	Nuclear factor kappa B
GPR109a	G-protein-coupled receptor 109A
IL-10	Interleukin-10
MAPK	Mitogen-activated protein kinase
PI3K	Phosphatidylinositol 3-kinase
ZO-1	Zonula occludens-1
NLRP3	NOD-like receptor protein 3
TLR4	Toll-like receptor 4
SREBP-1c	Sterol regulatory element-binding protein 1c
WAT	White adipose tissue
UCP1	Uncoupling protein 1
PPARγ	Peroxisome proliferator-activated receptor gamma
PGC-1α	Peroxisome proliferator-activated receptor gamma coactivator 1-alpha
β3-AR	β3-adrenergic receptor
p38 MAPK	P38 mitogen-activated protein kinase
NAFLD	Non-alcoholic fatty liver disease
4-HPAA	4-hydroxyphenylacetic acid
ILC3	Type 3 innate lymphoid cell
IL-22	Interleukin-22
CD36	Cluster of differentiation 36
NPC1L1	Niemann-Pick C1-like 1
FABP	Fatty acid-binding protein
AMPK	AMP-activated protein kinase
IAA	Indole-3-acetic acid
IAld	Indole-3-carbaldehyde
IPA	Indole-3-propionic acid
FGF19	Fibroblast growth factor 19
CYP7A1	Cholesterol 7α-hydroxylase
GLP-1	Glucagon-like peptide-1
CB1/CB2	Classical cannabinoid receptors 1/2
GPR119	G-protein-coupled receptor 119
GPR10	G-protein-coupled receptor 10
GPR55	G-protein-coupled receptor 55
GPR18	G-protein-coupled receptor 55
FAAH	Fatty acid amide hydrolase
Th1	T helper 1 cell
Th17	T helper 17 cell
5-AVA	5-Aminovaleric acid and other metabolites
FMT	Fecal microbiota transplantation
RCTs	Randomized controlled trials
